# Polarization and migration in the zebrafish posterior lateral line system

**DOI:** 10.1371/journal.pcbi.1005451

**Published:** 2017-04-03

**Authors:** Hildur Knutsdottir, Cole Zmurchok, Dhananjay Bhaskar, Eirikur Palsson, Damian Dalle Nogare, Ajay B. Chitnis, Leah Edelstein-Keshet

**Affiliations:** 1 Department of Mathematics, University of British Columbia, Vancouver, British Columbia, Canada; 2 Department of Biology, Simon Fraser University, Burnaby, British Columbia, Canada; 3 Division of Developmental Biology, Eunice Kennedy Shriver National Institute of Child Health and Human Development, Bethesda, Maryland, United States of America; Northeastern University, UNITED STATES

## Abstract

Collective cell migration plays an important role in development. Here, we study the posterior lateral line primordium (PLLP) a group of about 100 cells, destined to form sensory structures, that migrates from head to tail in the zebrafish embryo. We model mutually inhibitory FGF-Wnt signalling network in the PLLP and link tissue subdivision (Wnt receptor and FGF receptor activity domains) to receptor-ligand parameters. We then use a 3D cell-based simulation with realistic cell-cell adhesion, interaction forces, and chemotaxis. Our model is able to reproduce experimentally observed motility with leading cells migrating up a gradient of CXCL12a, and trailing (FGF receptor active) cells moving actively by chemotaxis towards FGF ligand secreted by the leading cells. The 3D simulation framework, combined with experiments, allows an investigation of the role of cell division, chemotaxis, adhesion, and other parameters on the shape and speed of the PLLP. The 3D model demonstrates reasonable behaviour of control as well as mutant phenotypes.

## Author summary

Collective migration of a group of cells plays an important role in the development of an organism. Here we study a specific example in the zebrafish embryo, where a group of about 100 cells (the posterior lateral line primordium, PLLP), destined to form sensory structures, migrates from head to tail. We model the process from the initial polarization to the migration, with a focus on how tissue polarity could arise. Using a 3D deformable-ellipsoid cell-based simulation, we explore the effects of cell-cell, cell-substrate, and cell-chemical interactions. We discuss drag forces experienced by cells and what that implies about the inherent active motion of both leading and trailing cells. The model allows us to test how each of several biological parameters affects the shape, size, effective migration and speed of migration. A subsequent study will be aimed at understanding the formation and deposition of neuromasts.

## Introduction

Collective cell migration has emerged as an important topic for research, combining biological experiments, computational biology and theoretical approaches. Key problems to be addressed include (1) How do cells maintain cohesion and directionality while migrating over long distances relative to cell and/or cell-cluster diameters? (2) What forms the guidance cues that directs cells to their targets? (3) How does cell division, active crawling, adhesion, and mechanical transduction interface with chemical signalling in cell collectives? (4) How do intra and intercellular signalling affect differentiation and distinct roles of leading and trailing cells?

Progress in exploring such questions has been most rapid in systems that are amenable to experimental probing. Unlike single-cell research that started decades ago by tracking isolated cells, studying cell collectives has mandated visualization of in vivo systems, with cells migrating or carrying out complex patterns of behaviour inside a living organism. In vitro and/or computational models also contribute to an increased understanding.

Among such systems, the zebrafish (*Danio rerio*) embryo has the advantages of (a) embodying vertebrate biology (placing it closer to mammalian systems than *Drosophila* or *C. elegans*) (b) being optically transparent (c) providing a number of cell behaviours and chemical signalling that can be related to other biological systems.

Here we focus on one developmental aspect of zebrafish development, the migration of the posterior lateral line primordium (PLLP) that eventually deposits neuromasts in its wake. (This system is responsible for generating sensory hair cells that respond to fluid flow along the lateral line organ.) In this paper we address the initial phases of development of this system, which provides an opportunity for studying basic questions in development.

The migrating PLLP is a dynamic structure, with changes in behaviour and cell morphology across the primordium. The PLLP consists of more than 100 cells that migrate together, along the length of the zebrafish embryo from head to tail (approximately 22-48 hours post-fertilization). The PLLP completes its migration over the course of a day, migrating around 2.3 mm [1]. During migration, the cells in the trailing region re-organize to deposit clusters (neuromasts) in their wake, a process that we consider in a future study.

The changes in cell behaviour across the PLLP are thought to be determined by a number of signalling networks. The PLLP is dominated by Wnt and FGF signalling. Cells at the front (rear) of the PLLP are dominated by WntR (FGFR) activity. [2, 3]. Through a mutually inhibitory signalling network, Wnt and FGF signalling is responsible for dividing the PLLP into the leading and trailing zones. Moreover, the Wnt and FGF signalling network modulate chemokine receptor expression in the leading and trailing zones, with the receptor CXCR4b expressed largely in the leading zone, and CXCR7b expressed in the trailing zone [4]. The PLLP migrates on a surface of cells that express a stripe of the chemokine CXCL12a (also known as SDF-1a). [5–7]. In addition, the leading and trailing cells cooperate to organize migration of the PLLP. The leading cells are responsible for following chemokine cues, while the trailing cells undergo chemotaxis toward the FGF ligand produced by the cells in the leading zone [8].

In order for a normal developmental sequence to take place, the cell mass in the PLLP must polarize and select a direction for migration. Failure of any part of the underlying chemical signalling, and/or disruptions in the mechanical properties give rise to mutants with specific migration or other defects, providing an opportunity to tease apart some of the underlying interactions.

Understanding the PLLP from experiments and verbal arguments alone is challenging. Theoretical analysis and computational cell-based simulations allow us to ask how a given hypothesis or set of hypotheses fare based on experimental observations in the literature.

Here we address a number of key questions:

1. Can Wnt-FGF signalling alone give rise to the initial polarization of the primordium into distinct leading and trailing domains?
2. How do the receptor-ligand signalling parameters and cells’ sensitivities to signalling levels affect the proportion of the tissue that develops into Wnt and FGF signalling domains?
3. Are the proposed interactions with the chemokine CXCL12a sufficient to explain the migration of the primordium?

Some of these questions have been partially addressed in previous modelling papers [8–12]. These models have examined the interactions of the PLLP with CXCL12a in migration [9], the migration and neuromast deposition [10], the mechanical conditions for efficient migration [11], the outcome of laser ablation of parts of the PLLP [8], and a flocking model for the cells in the PLLP [12]. All efforts contribute to piecing together aspects of the system, though a comprehensive simulation of the entire process is yet to be proposed. We compare these efforts to our own in the Discussion.

We first define the modelling framework and explain the computational system. Then we explore the polarization of the tissue, the chemical signalling, and how migration interfaces with signalling. We demonstrate that the model accounts for experiments and mutant types.

### Biological background

In this section, we describe additional biological background for our investigation.

The PLLP consists of more than 100 cells (≈4-5 cells wide) when migration is initiated, which migrate about 2.3 mm down the horizontal myoseptum during the time interval 22-48 hours post-fertilization [1]. Cells in the trailing region re-organize into rosettes, are periodically deposited and later develop into neuromasts, to form sensory organs of the posterior lateral line. The cells at the leading region are flatter and have a mesenchymal morphology [2].

The changes in cell morphology and behaviour across the PLLP is thought to be regulated by antagonistic Wnt-FGF signalling network [2–4]. The leading zone of the PLLP is characterized by high-levels of WntR activity while the trailing zone is dominated by FGFR activity. It is not known which Wnt ligand is involved although recent experimental work by members of our group (DDN and AC) implicates Wnt10a. More research has been done on the downstream Wnt signalling network [4]. WntR activity at the front of the PLLP promotes the expression of FGF3 and FGF10 ligands, as well as *sef*, considered to inhibit FGFR activity. Consequently, FGF pathway activation is inhibited in the leading cells, and the secreted FGF ligand can redistribute towards the rear. Activation of FGF signalling in the trailing domain subsequently drives the expression of Dkk1, a diffusible inhibitor of Wnt signalling [4]. In this way, Wnt and FGF signalling domains are established in the leading and trailing zones of the PLLP.

Migration is dependent on chemokine signalling across the primordium, and is guided by a strip of CXCL12a, a chemokine that is produced by superficial muscle pioneer cells of the horizontal myoseptum, has limited diffusion on the surface of this layer, and determines the PLLP’s migration path [5–7]. Without CXCL12a the PLLP stalls. However, directional information is not encoded by CXCL12a, whose expression level is uniform. Instead, the differential expression of two chemokine receptors, CXCR4b and CXCR7b, across the length of the PLLP guides migration. Similar to the Wnt-FGF polarity, the expression of CXCR4b dominates in the leading end while expression of CXCR7b dominates in the trailing zone with some overlap [5, 7]. Mechanisms that determine the differential expression of chemokine receptors in the PLLP are linked to the development of the polarized Wnt-FGF signalling, but are as yet incompletely understood [4, 10].

Cells expressing CXCR4b respond to CXCL12a with protrusive and migratory behaviour. The cells expressing CXCR7b rapidly internalize and degrade CXCL12a. It has been proposed that this sets up a local gradient of CXCL12a that ensures unidirectional migration of the PLLP [9]. It was also suggested that the absolute level of CXCL12a serves as cue [8]. Moreover, recent laser ablation and cutting experiments in [8] revealed that the trailing cells undergo chemotaxis toward FGF ligand secreted by the leading PLLP cells. Evidence for this stems from several experiments described in [8]: (1) when trailing cells are physically separated from the leading cells by laser ablation, trailing cells move toward and eventually join with leading cells despite unpolarized expression of the chemokine receptor CXCR4b in the trailing fragment. (2) This movement is inhibited by treatment with the FGF inhibitor SU5402, suggesting that FGF signaling is essential for this process. (3) Furthermore, an isolated trailing cell cluster generated by laser ablation can be induced to migrate directionally by placing a bead soaked in recombinant FGF3 either ahead of or behind the cells. This suggests that while the leading and trailing cells coordinate migration by inducing a local CXCL12a gradient, the leader cells respond to chemokine cues and the trailing cells migrate toward the leading cells [8].

### Modelling framework

#### Chemical signalling

We first propose a simple chemical signalling model and use it to address the initial stage of polarization of the PLLP into a WntR active front and an FGFR active rear. While the biological literature provides evidence that mutual inhibition between WntR and FGFR activities is involved, it remains to be determined how that inhibition is mediated, and what details control the boundary between the two domains.

**Fig 1:**
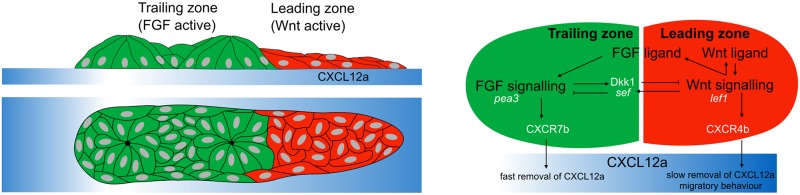
The geometry and signaling of the posterior lateral line primordium (PLLP). Left: A sketch of the PLLP showing side and top-down views, with leading (red) and trailing (green) cells on a stripe of CXCL12a. Right: A schematic diagram of signalling in the PLLP, showing the mutual inhibition between FGFR and WntR signalling. (Black text denotes model components, white text aides in interpretation of experiments.) FGF signaling inhibits Wnt signaling by determining expression of Dkk1; Wnt signaling cells express the gene *sef* that inhibits FGF signaling. WntR active cells are sources of both FGF and Wnt ligands. Experimentally, *pea3* and *lef1* expression levels are used to identify FGF and Wnt signalling, respectively. The WntR-FGFR activity polarization sets up chemokine polarization (CXCR4b vs CXCR7b). In our model, this leads to the creation of a gradient of CXCL12a that enables directed migration of the PLLP.

As inputs to the model, we use several assumptions. (1) A given cell is considered to be a WntR (respectively FGFR) active cell if the level of cell surface Wnt (respectively FGF) receptors is high (relative to some baseline level). (2) It is known that there is a mutually inhibitory interaction between FGFR and WntR activities [13]. Our purpose is to propose a reasonable receptor-based mechanism for this mutual inhibition. Consequently, we postulate that bound Wnt (respectively FGF) receptors on the cell surface activate signalling pathways inside the cell that lead to inhibition of expression of receptors of the other signalling type. Activation of Wnt signaling determines the expression of factors that directly (*sef*) or indirectly (*dusp6*) interfere with efficacy of signaling by FGF receptors. In general mutual antagonism could be based on mutual interference by one type of bound receptor with the surface presentation of the other type, see e.g., [4]. As a result, bound FGF receptors on a cell contribute to inhibition of Wnt receptors on its surface and vice versa, see Fig 1.

#### Cell-surface receptor levels

We represent the state of each cell by the number and type of receptors (Wnt vs. FGF) on the cell surface. Cells that have mostly Wnt receptors on their surface are denoted “Wnt expressing cells” and similarly for FGF. In order to allow for cell commitments to evolve with time, we implement mutually inhibitory interactions between Wnt and FGF signalling as described below.

Let *R*(*t*) be the level of expression of a given receptor type on the surface of a given cell (for example, in units of nM). Then we model the rate of change of *R*(*t*) by

$$\begin{array}{c} \frac{dR}{dt}=\text{presentation}\;\text{rate}-\text{endocytosis}\;\text{rate}. \end{array}$$ (1)

Both the presentation rate and the endocytosis rate are regulated by signalling networks. Here we assume the simplest model, in which mutual FGF-Wnt inhibition affects the presentation rate of the antagonist receptors (as in, for example, [14]), but results are very similar for regulation via damping of presentation or accentuation of endocytosis rate or both simultaneously (see, e.g. [15], where analogous small positive and negative feedback circuits are analyzed in detail). It is also possible to include positive feedback of signalling on the rate of presentation of its own receptor type. Save for a few specific scenarios, explained in Supporting Information S3 Text. Receptor positive feedback, results are largely similar.

We define *F*_*R*_(*x*, *t*), *W*_*R*_(*x*, *t*) to be the concentration of FGF and Wnt cell surface receptors (in units of nM), and *F*_*B*_(*x*, *t*), *W*_*B*_(*x*, *t*) to be the concentration of bound receptors at position *x*. (In general, *x* denotes a position within the PLLP in the full 3D model. In the 1D model reduction, we average across the width and thickness of the PLLP, and restrict attention to variations of signalling levels across its length. In that case, *x* represents position along the primordium 0 ≤ *x* ≤ *L*, where *x* = *L* is the front of the primordium and *x* = 0 is the back). The respective FGF and Wnt ligands are denoted *F*(*x*, *t*), *W*(*x*, *t*).

Receptors can be in either unbound (“free”) or ligand-bound (“*B*”) form on the cell surface. The total level of receptors, *F*_*R*_, *W*_*R*_ satisfy

$$\begin{array}{c} F_{R}\left(t\right)=F_{B}\left(t\right)+F_{free}\left(t\right),\;W_{R}\left(t\right)=W_{B}\left(t\right)+W_{free}\left(t\right). \end{array}$$ (2)

The total population of receptors on the cell surface, *F*_*R*_, *W*_*R*_, is used as a model readout of the “state” of the cell: when *F*_*R*_ ≫ *W*_*R*_, for example, the cell is considered to be an FGF-expressing cell (and vice versa for Wnt-expressing cells). The mutually inhibitory signalling within a cell is assumed to depend on *F*_*B*_, *W*_*B*_ based on the idea that only the bound receptors signal to downstream intracellular regulatory networks governing receptor synthesis and presentation.

**Fig 2:**
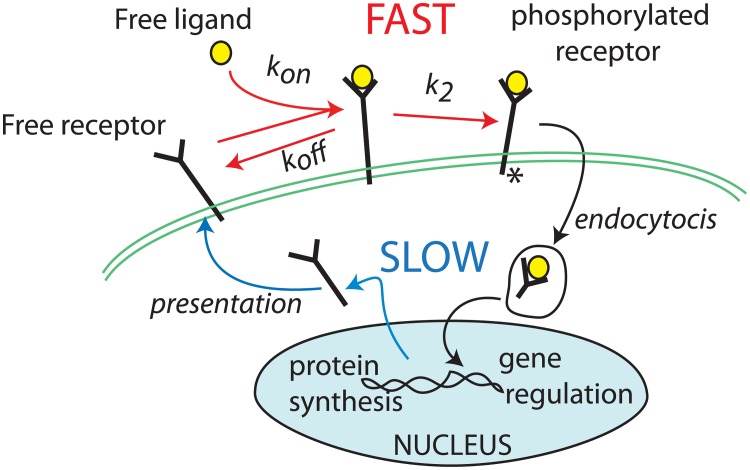
Ligand-receptor dynamics forms a basis for our model. FGF or Wnt receptors are synthesized, presented on the cell surface, and, after binding to ligand, phosphorylated and internalized by endocytosis. Here we assume that ligand-receptor binding is rapid on the timescale of such receptor dynamics, so that we can approximate the bound receptor level by Eq (3).

We assume that ligand binding to receptors is fast on the timescale of the receptor presentation and endocytosis rates, so that the bound receptors are in quasi steady state (QSS) with the available ligand. (See Fig 2). Then, by standard Michaelis-Menten kinetics,

$$\begin{array}{c} F_{B}\left(t\right)\approx \frac{F_{R}\left(t\right)F\left(t\right)}{K_{F}+F\left(t\right)},\;W_{B}\left(t\right)\approx \frac{W_{R}\left(t\right)W\left(t\right)}{K_{W}+W\left(t\right)}, \end{array}$$ (3)

where *K*_*F*_, *K*_*W*_ are equilibrium binding constants that are related to the rates of association and dissociation of the ligand-receptor complex (Fig 2 and Eqn. (12e) in Supporting Information S1 Text. Model equations, derivation and scaling).

Based on Eq (1) and assumptions above, the evolving levels of Wnt and FGF cell surface receptors are modeled as:

$$\begin{array}{cc} \frac{dF_{R}}{dt} & =E_{F}\left(W_{B}\right)-r_{F}F_{R} \end{array}$$ (4a)

$$\begin{array}{cc} \frac{dW_{R}}{dt} & =E_{W}\left(F_{B}\right)-r_{W}W_{R} \end{array}$$ (4b)

where receptor endocytosis rates, *r*_*F*_, *r*_*W*_ are assumed constant, and rates of presentation, *E*_*F*_(*W*_*B*_), *E*_*W*_(*F*_*B*_) of the receptors are modified by mutually inhibitory feedback. Note that we neglect the delay between binding of receptors and inhibition of receptor presentation pathways. The rates *E*_*F*_(*W*_*B*_), *E*_*W*_(*F*_*B*_) are decreasing functions of bound antagonist receptors. In particular, we take simple, commonly used decreasing Hill functions typical for such inhibitory terms, e.g., [16]:

$$\begin{array}{c} E_{F}\left(W_{B}\right)=\frac{E_{F,1}}{1+{\left(W_{B}/W_{0}\right)}^{n}},\;E_{W}\left(F_{B}\right)=\frac{E_{W,1}}{1+{\left(F_{B}/F_{0}\right)}^{m}}. \end{array}$$ (4c)

Here *E*_*i*,1_ is the basal rate of receptor presentation when there is no inhibition, and *F*_0_, *W*_0_ are the “IC_50_” inhibition parameters with units of receptor density. For example, when bound FGF receptors are at the level *F*_*B*_(*x*, *t*) = *F*_0_, then the Wnt receptor presentation rate is reduced to 50% of its basal level. *n*, *m* ≥ 2 are Hill coefficients. The larger these values, the sharper the switch turning off the presentation of receptors. We typically take *n* = *m* = 5, which slightly increases the regime of bistability of the system compared to the case *n*, *m* ≈ 2, but similar behavior is obtained for *n* ≠ *m* and for smaller values of these Hill coefficients. The limit *n* → ∞ leads to analytic insights, as noted in [17]. In brief, negative feedback is proportional to decreasing Hill functions of the form

$$\begin{array}{c} g\left(c\right)=\frac{1}{1+c^{m}},\;0\leq g\left(c\right)\leq 1 \end{array}$$

where *c* is a dimensionless quantity (ratio of bound receptor level to IC_50_ parameter). As expected (see e.g. [14]), this type of mutually-inhibitory mechanism can, under appropriate conditions, give rise to subdivision of the tissue into two domains.

Our Wnt-FGF receptor model is a minimal model that can set up tissue polarization. Other variants could include FGF-bound receptors up-regulating Wnt receptor endocytosis, as well as additional positive feedback (e.g. from FGF bound receptors to FGF receptor presentation, e.g. see Supporting Information S3 Text. Receptor positive feedback) [4] or other signalling cascades. In practice, given the lack of detailed data for such terms, we chose to use the minimal model variant at present. Furthermore, we concentrate on qualitative, rather than quantitative aspects of the model results, since parameter values and biochemical details are not available to us.

#### FGF and Wnt ligand concentration

The ligands diffuse (with diffusion coefficients *D*_*F*_, *D*_*W*_), bind to free receptors (at rates *k*_*F,on*_ and *k*_*W,on*_), and have some turnover rates *δ*_*F*_ and *δ*_*W*_. When the ligand concentrations are normalized by baseline (steady state) values, the dimensionless equations for ligand concentrations are:

$$\begin{array}{cc} \frac{\partial F}{\partial t} & =D_{F}\frac{\partial^{2}F}{\partial x^{2}}-\delta_{F}F-\kappa_{F}F_{R}\frac{F}{1+F}+\rho_{F}W_{R}\frac{W}{1+W} \end{array}$$ (5a)

$$\begin{array}{cc} \frac{\partial W}{\partial t} & =D_{W}\frac{\partial^{2}W}{\partial x^{2}}-\delta_{W}W-\kappa_{W}W_{R}\frac{W}{1+W}+\rho_{W}W_{R}\frac{W}{1+W} \end{array}$$ (5b)

where

$$\begin{array}{c} D_{i}=\frac{D_{i}}{L^{2}},\;\kappa_{F}=k_{F,\text{on}}F_{1},\;\kappa_{W}=k_{W,\text{on}}W_{1}, \end{array}$$ (5c)

and

$$\begin{array}{c} \rho_{F}=\frac{p_{F}W_{1}}{K_{F}},\;\rho_{W}=\frac{p_{W}W_{1}}{K_{W}}. \end{array}$$ (5d)

(See details of scaling in Supporting Information S1 Text. Model equations, derivation and scaling.) Here *D*_*i*_ = *D*_*F*_, *D*_*W*_ are rates of diffusion of the FGF and Wnt ligands, *δ*_*F*_, *δ*_*W*_ are the rates of decay of the ligands due to nonspecific degradation in the PLLP, *k*_*i*, on_ are rates of removal by binding to ligand-specific receptors of the given type and *F*_1_, *W*_1_ are steady state receptor levels when there is no negative feedback. The terms, $\rho_{F}W_{R}\frac{W}{1+W}$ and $\rho_{W}W_{R}\frac{W}{1+W}$, in Eq (5) model the production of FGF and Wnt ligands respectively by WntR active cells. (See the derivation of these terms in Supporting Information S1 Text. Model equations, derivation and scaling.)

#### Discrete cell model

As before, we aim to obtain a qualitatively reasonable representation of the PLLP, since many details of the cell behaviour and interactions, and most quantitative rates and parameters are as yet unknown. Hence, all our model parameters and assumptions should be viewed as ballpark reasonable estimates.

The detailed implementation of our 3D discrete cell-based model is described in the Materials and Methods section. Briefly, cells experience mechanical forces from (1) mutual interactions (very-short-range repulsion for excluded volume effects and short-range adhesion to neighboring cells; both these forces depend on the distance between neighbor surfaces along a line segment joining neighbor cell centers, (2) chemotaxis up gradients of CXCL12a and FGF, (3) drag forces, and (4) adhesion to the substrate (normal to the direction of motion, and hence only affecting the flatness of the PLLP, but not its motion). The balance of these forces determines cell shape and movement. Model cells are both sources and sinks of Wnt and FGF ligands (tracked using reaction-diffusion equations solved on a 3D Cartesian grid above the plane of the substrate). The slow diffusion of the CXCL12a chemokine in the plane of the substrate is tracked in 2D. We assume that secretion and uptake of ligands in a small volume element (voxel) is proportional to local cell-surface area in the given voxel.

**Fig 3:**
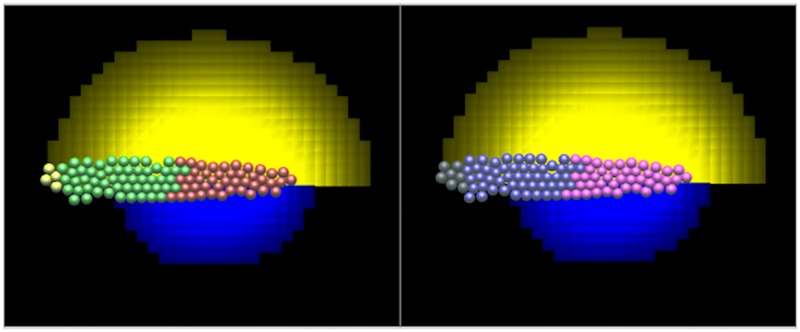
Illustration of the simulations using the 3D deformable ellipsoid cell-based model. View from above of a 1-cell layer representing the PLLP. The stripe of CXCL12a would be directly underneath the PLLP, not explicitly shown. For visualization purposes, the Wnt (blue) and FGF (yellow) ligands are shown as a pair of clouds in each split-image. Left panel: cells are colored based on receptor expression level (red for high Wnt and green for high FGF receptor levels, *W*_*R*_, *F*_*R*_). FGFR and WntR expressing cells are colored yellow. Right panel: cells colored by their *bound* receptor levels (pink for high Wnt and purple for high FGF bound-receptor levels, *W*_*B*_, *F*_*B*_). In our model we interpret the latter as the Wnt or FGF *signalling levels*. Cells in the back of the PLLP express FGF receptors but do not signal since FGF ligand is so low that most FGF receptors are unbound. Grey or yellow cells at the back of the PLLP are those that are not yet committed to being either WntR or FGFR active cells. Results from the full 3D model after 30 min of simulation time (before the onset of migration).

The receptor levels *W*_*R*_, *F*_*R*_ of each cell are computed according to Eq (4). We then assign those cells with *W*_*R*_ > *W*_*thresh*_ to be a *Wnt receptor (WntR) expressing cell* (shown red in Fig 3) versus cells with *F*_*R*_ > *F*_*thresh*_ that we denote *FGF receptor (FGFR) expressing cells* (colored green). If both of these conditions hold then the cells are both FGFR and WntR expressing cells (colored yellow). If neither of these conditions hold, the cells are taken to be of undetermined fate (colored grey). The threshold values *W*_*thresh*_, *F*_*thresh*_ are assumed to be 90% of the steady state receptor levels (denoted *W*_1_, *F*_1_, see Table A in Supporting Information S6 Text. Parameter Estimation and Values). The results in Fig 3 are from simulations using the full 3D model but at an early time point (30 minutes of simulation time) when the PLLP has polarized but migration has not started.

Wnt (respectively FGF) expressing cells act as sinks that bind the given ligand. We assume that all Wnt receptor expressing cells secrete FGF and Wnt ligands. To compare our results to experimental observations, we also show the level of bound receptors, *W*_*B*_ and *F*_*B*_ on our simulated cells, in Fig 3 right panel. WntR active cells, *W*_*B*_, are shown in pink, FGFR active cells, *F*_*B*_, cells are shown in purple and cells that are neither are shown in grey.

A stripe of CXCL12a is produced from head to tail by underlying muscle cells, undergoes slow diffusion in the plane of the substrate, and is degraded by PLLP cells as they migrate above it. Its concentration is simulated along the 2D surface on which the PLLP moves, according to the equation:

$$\begin{array}{c} \frac{\partial C}{\partial t}=D_{C}\nabla^{2}C+\alpha -\delta_{C}C-\kappa_{CW}C\Omega_{W}-\kappa_{CF}C\Omega_{F} \end{array}$$ (6)

where *D*_*C*_ is the diffusion rate of CXCL12a, *α* is a constant production of CXCL12a along the centre line of the domain, *δ*_*C*_ is the uniform nonspecific degradation of CXCL12a, *κ*_*CW*_ (*κ*_*CF*_) is the breakdown of CXCL12a by cells expressing WntR (FGFR/undetermined). *Ω*_*W*_ (*Ω*_*F*_) represents the local fraction of cells expressing WntR (FGFR/undetermined), with 0 ≤ *Ω*_*W*, *F*_ ≤ 1. We choose the diffusion, decay and production rates such that in the absence of the primordium there is a steady state level of CXCL12a along the centre line of the domain.

Cells expressing FGFR and WntR respond to gradients of FGF and/or CXCL12a, provided the concentrations of the ligand(s) are above some detectable level. In that case, the cell orients towards a vector sum of the gradients, and moves with constant speed (chemotactic force is assumed to have a constant magnitude, independent of ligand type and gradient steepness). If ligand is undetected, the cell’s axis of polarity (and hence direction of motion) can reorient randomly by up to 20° in a time step of 1 min (see Table A, in Supporting Information S4 Text. Discrete cell model, for parameter values).

## Results

### Tissue subdivision into leading and trailing zones

Our initial step was to explore mechanisms for the subdivision of the tissue into a Wnt-signalling leading domain and a FGF-signalling trailing domain. We specifically sought to determine how a spatially graded parameter, such as the steady state level of the Wnt receptors (*W*_1_), across the length of the primordium can set up this domain subdivision, and where the domain boundary would be positioned. We first explore this from a mathematical perspective, linking the outcome to underlying biochemistry, and then show the results numerically. We employ a 1D model simplification followed by the full 3D simulation for the PLLP.

#### Theoretical considerations

The separation of timescales of ligand-receptor binding (fast) and receptor presentation/endocytosis (slow), allows us to use Michaelis-Menten kinetics to simplify Eq (4) so that the model is recast in terms of the total level of cell-surface receptors, *F*_*R*_(*x*, *t*), *W*_*R*_(*x*, *t*) and the ligand concentrations *F*(*x*, *t*), *W*(*x*, *t*).

In absence of the negative feedback, the steady state receptor level is (*F*_*R*_, *W*_*R*_) = (*E*_*F*,1_/*r*_*F*_, *E*_*W*,1_/*r*_*w*_)≡(*F*_1_, *W*_1_). We scale the variables by these steady state levels. Under this scaling, Eq (4) can be rewritten in the form

$$\begin{array}{cc} \frac{dF_{R}}{dt} & =r_{F}\left[\frac{1}{1+{\left(W_{R}/\omega \right)}^{m}}-F_{R}\right] \end{array}$$ (7a)

$$\begin{array}{cc} \frac{dW_{R}}{dt} & =r_{W}\left[\frac{1}{1+{\left(F_{R}/\phi \right)}^{n}}-W_{R}\right] \end{array}$$ (7b)

where

$$\begin{array}{c} \phi =\frac{F_{0}}{F_{1}}\left(\frac{K_{F}+F}{F}\right),\;\omega =\frac{W_{0}}{W_{1}}\left(\frac{K_{W}+W}{W}\right) \end{array}$$ (7c)

(see Supporting Information S1 Text. Model equations, derivation and scaling, and note that *F*_*B*_(*x*, *t*), *W*_*B*_(*x*, *t*) have been eliminated from the equations). In Eq (7), the space and time dependent quantities *ϕ*(*x*, *t*), *ω*(*x*, *t*) are related to underlying molecular biochemistry such as ligand concentrations, binding constants, and signalling IC_50_ parameters. In the Supporting Information S3 Text. Receptor positive feedback, we also discuss a slightly expanded variant of this model in which positive feedback is included in both equations.

**Fig 4:**
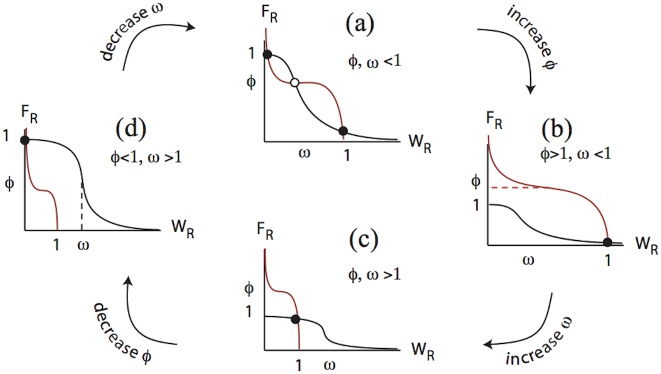
Analysis of mutual inhibition dynamics leads to model insights. The scaled mutual inhibition Eq (7) can have one of four possible behaviours represented by nullclines in the *W*_*R*_ *F*_*R*_ phase plane. (*F*_*R*_ nullcline in black, *W*_*R*_ nullcline in red.) (a) Bistability, in which either a high Wnt receptor (*W*_*R*_) or high FGF (*F*_*R*_) receptor expressing state can result, depending on initial conditions. (b) A Wnt-receptor expressing state always result, (c) a coexistence-state, with both Wnt and FGF receptors expressed, and (d) an FGF only receptor expression state exists. When the Hill coefficients *n*, *m* are large, the steady states (appropriately scaled) occur approximately at a subset of the points {(1, 0), (1, 1), (0, 1)} and transitions between the four qualitative outcomes displayed in this figure can be summarized by simple inequalities in terms of aggregate quantities *ϕ* and *ω*, see Eq (7c).

The behavior of Eq (7) can be understood by sketching nullclines of the system (curves that depict loci for *dF*_*R*_/*dt* = 0, and *dW*_*R*_/*dt* = 0) as shown in Fig 4. Steady states are points of intersection of those nullclines. This is done most easily in the “sharp switch” limit of these equations where Hill functions are approximated as on-off switches (*n*, *m* → ∞), as the analysis of steady states is particularly simple [17]. Indeed, we have that steady states will be at intersections of piece-wise constant nullclines in this limit, namely,

$$\begin{array}{cc} F_{R}\text{ nullcline: }F_{R}=1\text{ if }W_{R}<\omega ; & F_{R}=0\text{ otherwise},\\ W_{R}\text{ nullcline: }W_{R}=1\text{ if }F_{R}<\phi ; & W_{R}=0\text{ otherwise}. \end{array}$$

We can analyze the spectrum of behavior of Eq (7) for constant values of the quantities *ϕ* and *ω*, and then describe what happens when these quantities are time and space dependent. In particular, as concentrations and other biochemical parameters vary across the primordium, both *ϕ* and *ω* will also vary across the domain. In principle, several possibilities are theoretically possible for the mutual-inhibition model in the spatial context. For example, opposed gradients of *ϕ* and *ω* could lead to a transition from Wnt-dominated front, to FGF dominated rear with or without an overlap region in the middle [14]. This would occur if either *ϕ* or *ω* cross their respective thresholds (*ϕ* = 1, *ω* = 1) somewhere in the domain (a coexistence state, which implies a smooth transition zone between front and back requires that *ϕ* > 1, *ω* > 1 over some region in the middle of the domain). Within the context of our simplified model for the PLLP, this scenario cannot happen because opposite gradients of *ϕ* and *ω* do not occur. (This stems from the fact that both Wnt and FGF ligands are graded from front to back.) As we shall discuss in the next section, the domain always evolves to the bistable state (Fig 4a) with *ϕ* < 1, *ω* < 1 everywhere. The front becomes *W*_*R*_ dominated, and the rear becomes *F*_*R*_ dominated, but the transition zone is sharp, and determined by a separatrix (boundary of two basins of attraction). Future models that consider the role of Dkk1 and/or other effectors that vary spatially might amend this feature of the model.

#### The 1D spatial simulation

We implemented the signalling model (Eqs (5) to (7)) in one spatial dimension, with default parameters estimated from the literature, where possible (Table A in Supporting Information S6 Text. Parameter Estimation and Values, and details in Materials and Methods). To do so, we scaled the length of the primordium to *L* = 1, with rear and front at *x* = 0 and *x* = 1, respectively. To understand how the signalling domains might form, we first considered initial conditions in the “coexistence” state (Fig 4) with *W*(*x*, 0) = 0.01, *F*(*x*, 0) = 0.005, *W*_*R*_(*x*, 0) = 0.1, and *F*_*R*_(*x*, 0) = 0.01. These represent small initial values of the scaled ligand and receptor levels (scaling previously discussed) that would tend towards the unique coexistence state over time. We assumed that the parameter *W*_1_ is initially graded across the PLLP (*W*_1_(*x*) = b*x*+0.03). Motivating this assumption is the experimental observation that the Wnt signalling dependent transcription factor *lef1*, has graded expression (highest at front). We then adjusted the rate of secretion of Wnt and FGF ligand to obtain biologically realistic signalling domains. Signalling domains form as expected. Note that zones readily form with many other types of initial bias, e.g., graded *W*_*R*_, (see Supporting Information S2 Text. Formation of zones with graded initial Wnt Receptor concentration) or flux of Wnt ligand from the right boundary.

**Fig 5:**
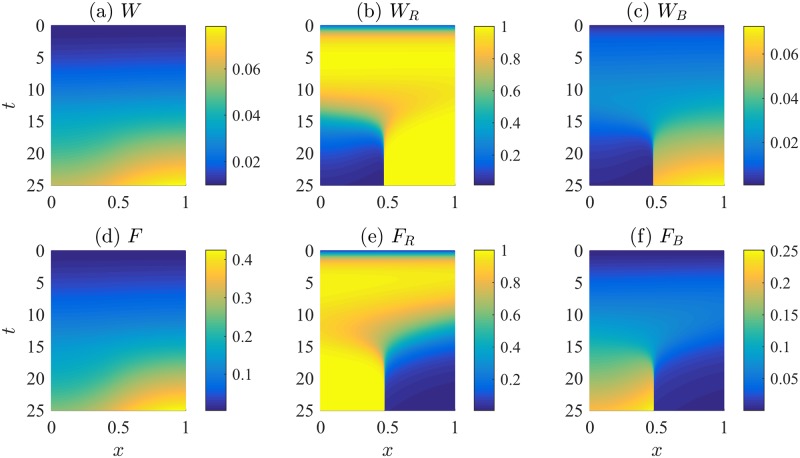
The Wnt-FGF mutual inhibition model predicts the formation of signalling domains in response to graded *W*_1_ across the primordium. Simulation kymographs for Wnt and FGF signalling. Initially, we assumed a gradient *W*_1_(*x*) = *bx* + 0.03 in the uncoupled Wnt steady state parameter, with *b* = 0.01. Time increases downwards; position across the PLLP is horizontal, with leading edge on the right. (a) Wnt ligand, *W*(*x*, *t*), (b) Wnt receptors, *W*_*R*_(*x*, *t*), (c) bound Wnt receptors, *W*_*B*_(*x*, *t*), (d) FGF ligand, *F*(*x*, *t*), (e) FGF receptors, *F*_*R*_(*x*, *t*), and (f) bound FGF receptors, *F*_*B*_(*x*, *t*). Signalling domains form after a few minutes, with a sharp boundary between zones. Bound ligand concentrations are calculated using Eq (3). Parameters are as in Table A in Supporting Information S6 Text. Parameter Estimation and Values. Initial conditions: *W*(*x*, 0) = 0.01, *F*(*x*, 0) = 0.005, *W*_*R*_(*x*, 0) = 0.1, *F*_*R*_(*x*, 0) = 0.01.

**Fig 6:**
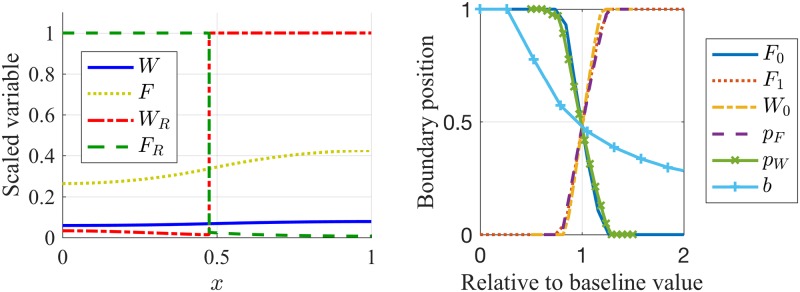
The position of the boundary between leading and trailing zones is parameter dependent. In the left panel, the concentration profiles of the ligand and receptor activity are plotted at *t* = 25 minutes (after formation of signalling domains), which reveals the formation of a sharp boundary between the leading and trailing zones. In the right panel, the boundary position between the leading and trailing zones is shown to be a function of model parameters. Parameter sweeps were conducted for a range of “IC_50_” inhibition parameters *F*_0_, *W*_0_, FGF steady state receptor levels *F*_1_, production of Wnt and FGF ligand *p*_*W*_, *p*_*F*_, as well as the slope of the *W*_1_ gradient across the PLLP, *b*. The vertical axis represents the fraction of the PLLP length that is FGF-receptor expressing at steady state. The horizontal axis represents the given parameters, scaled by their baseline values as listed in Table A in Supporting Information S6 Text. Parameter Estimation and Values.

Results of the 1D simulations are displayed in Fig 5. We show kymographs of the ligand and receptor levels of each type (Wnt, top, and FGF, bottom) across the 1D length of the tissue. After some transient behaviour that depends on initial conditions, we observe the formation of robust zones by roughly 20 minutes, with strong interfaces that persist. The shapes of the chemical profiles and receptor densities at *t* = 25 minutes are shown on the left panel of Fig 6. Both Wnt and FGF ligands are graded towards the front, with FGF ligand the sharper of the two. The receptor densities exhibit a sharp interface in the middle of the domain, as expected from the mutual-inhibition model.

We investigated the time evolution of the values of *ϕ* and *ω* over the domain. At *t* = 0, given the initial gradient in *W*_1_, every point in the domain satisfies *ϕ* > 1, *ω* > 1, implying a single coexistence (Wnt and FGF) steady state everywhere. The gradient in *W*_1_ induces a gradient in *ω*, which ensures that less Wnt activity at the front of the PLLP is required to inhibit FGF activity. As FGF and Wnt ligand is produced, *ω* and *ϕ* fall below 1, while *W*_*R*_ and *F*_*R*_ signalling levels evolve. First, as *ω* decreases below 1, the PLLP transforms to a Wnt-dominated system. Second, as *ϕ* drops below 1, the PLLP becomes a bistable system, with spatially varying *F*_*R*_ and *W*_*R*_ conditions. While the front is already locked into Wnt dominance, the rear is diverted from its Wnt-wards trajectory into a basin of attraction of FGF-dominance, and becomes *F*_*R*_ dominated. In the final state, a sharp zone boundary forms between front and rear, determined by parameters that define the separatrix in the bistable state.

#### Parameter dependence of the zone boundary

We next asked how various parameters in the signalling model affect the position of the zone boundary. Since the competition between Wnt and FGF signalling is biased by the ligand concentration, by ligand binding to receptors, and by the sensitivity of a given cell type to bound receptors of the opposite type, it stands to reason that any one of these parameters would affect the subdivision of the tissue into zones.

To investigate this dependence, we ran 1D simulations, as before, and identified the location of the sharp receptor interfaces for 20 values of each of the parameters *W*_0_, *F*_0_ (“IC_50_” inhibition parameters *F*_1_ (steady state FGF receptor level), *p*_*F*_, *p*_*W*_ (ligand secretion rates), and *b* (slope of initial *W*_1_ gradient). The boundary location is shown in Fig 6 as a function of each of these parameters. The top edge of the panel represents the leading edge (*x* = 1), while the bottom (*x* = 0) is the trailing edge. When the boundary position is 0, the entire primordium is dominated by Wnt receptor expression, and when the boundary position is 1, the entire primordium is dominated by FGF receptor expression. The horizontal axis gives the ratio of parameter value to the baseline value used to produce signalling domains as in the left panel of Fig 6. For example, to produce the signalling domains in the left panel of Fig 6, we choose *b* = 0.01, and find that the boundary position is approximately at *x* = 0.5. When this parameter is increased to *b* = 0.02, we know that the ratio between *b* and its baseline value is 2, and we can identify the boundary position from the light blue curve in Fig 6 to be approximately at *x* = 0.25.

As expected, increasing either the basal (uninhibited) steady-state FGF-receptor level, *F*_1_, or the “IC_50_” inhibition parameter *W*_0_ (which decreases the sensitivity of FGF receptor presentation to inhibition by Wnt) biases the cells towards FGF receptors and hence pushes the boundary towards the front while increasing *F*_0_ (which decreases the sensitivity of Wnt receptor presentation to inhibition by FGF) has the opposite effect. The effects of changing each of the aggregate parameters, *W*_0_, *F*_0_ or *F*_1_, on the boundary position between the leading and trailing zones matches our intuition about the signalling dynamics. For example, by increasing *F*_1_ or *W*_0_, we bias the primordium towards FGF receptor expression. If *F*_1_ increases, the parameter *ϕ* decreases in Eq (7). This reduces the total number of FGF receptors required to inhibit Wnt receptor expression, whence the primordium is biased toward FGF receptor expression. Similarly, if *W*_0_ increases, so does the parameter *ω*. This increases the total number of Wnt receptors required to inhibit FGF receptor expression, whence the primordium is biased toward FGF receptor expression. The secretion of Wnt and FGF ligand also influences the position of the boundary. *W* and *F* appear in *ω* and *ϕ*. Hence any changes in the secretion rate *p*_*W*_ and *p*_*F*_ will affect the number of signalling receptors required to inhibit receptors of the opposite type. Lastly, note that the steepness of the gradient of *W*_1_ (basal steady state level of Wnt receptors when there is no FGF inhibition) across the primordium can affect the boundary position. One observation is that as *b* → 0, *W*_1_(*x*)→0.03. In this case, no species can induce a gradient in *ϕ* or *ω* across the PLLP, whence no signalling domains form.

#### Formation of zones in 3D simulations

Having obtained zone partitioning in the 1D model, we next asked whether a similar subdivision into leading and trailing domains would take place in the full 3D cell-based model. Consequently, we ran the full 3D model with the basic parameter set, as described in the Materials and Methods section. In all cases, we initiate the system with 100 uncommitted cells, and no ligand of either type. To resolve transients in the initial shape, we first allow cells to adhere and interact with each other and with the center of the domain where the CXCL12a stripe is located. This leads to an elongated cluster. (In the absence of the added adhesion to the CXCL12a stripe, we found that the cell cluster is round). We then turn on the signalling dynamics with W_1_ graded across the length of the primordium (*W*_1_ = 0.03 + 0.006*x*, where here, *x* represent the distance from the leading front of the PLLP in units of *μ*m.) The characteristics of the cells (whether WntR expressing, FGFR expressing or uncommitted) are determined by the levels of FGF and Wnt receptors; an undetermined state occurs when both Wnt and FGF receptors are below some set threshold.

**Fig 7:**
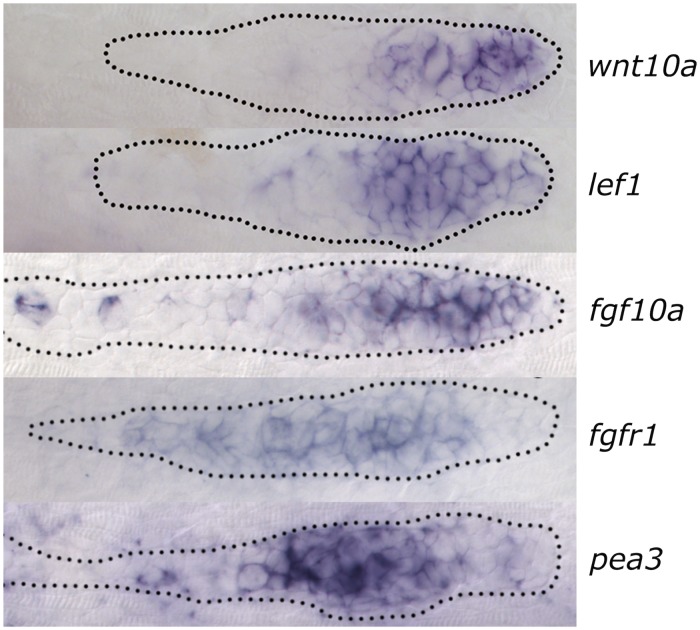
Experimentally observed Wnt and FGF expression and signalling levels. RNA in-situ hybridization showing expression at 32 hours post fertilization in the PLLP. From top to bottom these are: wnt10a (Wnt ligand), *lef1* (Wnt signalling), fgf10a (FGF ligand), fgfR1 (FGF receptors) and *pea3* (FGF signalling). The scale bar is 10*μ*m.

Results are shown on the left side of Fig 3 (with green for FGFR expressing cells, red for WntR expressing cells, yellow for both WntR and FGFR expressing cells and grey for uncommitted cells). (In all figures, “front” is on the right, “rear” on the left). We also show the Wnt ligand concentration profile (blue) and FGF ligand (yellow); both ligands are actually distributed throughout the 2D myoseptum on which the PLLP migrates (the *z* = 0 plane), but for visualization purposes, we show each as a split image. The right side of Fig 3 is from the same simulation as the left side but we used different colors to emphasize the distinction between *receptor expressing cells* and *signalling cells/bound receptors*. The pink cells on the right are the W_*B*_ cells, i.e. cells with high levels of Wnt ligand-bound Wnt receptor. The purple cells on the right are F_*B*_ cells, i.e. cells with high levels of bound FGF receptors. The grey cells on the right express FGF receptors but since the FGF ligand level and hence bound FGF receptors are very low, those cells are not considered FGF signalling cells. These results can be compared to experimental results where *lef1* is used as a marker for the Wnt signalling zone, *pea3* is used as a marker for the FGF signalling zone. Wnt10a is one of the candidates for the Wnt ligand contributing to this signalling network, FGF10a is one of the FGF ligands and the FGF receptors can also be labelled, see Fig 7.

**Fig 8:**
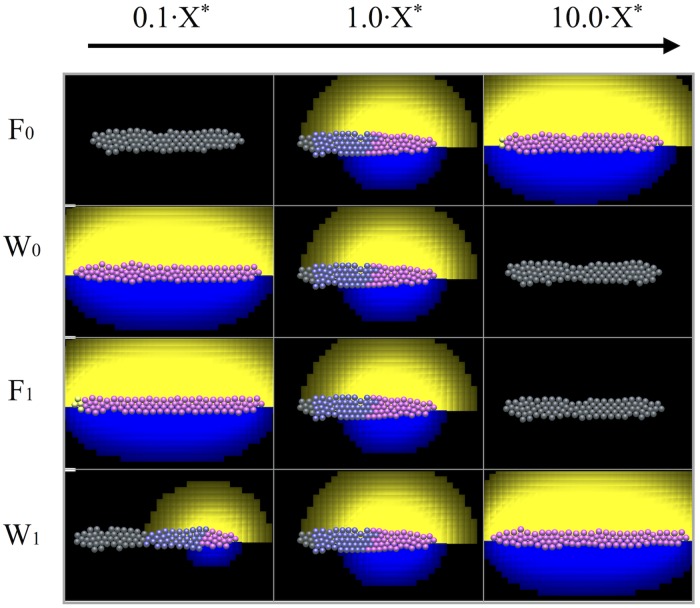
The position of the boundary between the leading and trailing zones is parameter dependent. The four parameters, *F*_0_, *F*_1_, *W*_0_ and *W*_1_ are altered. In the first column the baseline value (X*) has been multiplied by 0.1 and in the last column it has been multiplied by 10. Increasing *F*_0_ (top row) and *W*_1_ (bottom row) results in an increase in the size of the Wnt domain. Conversely, increasing *F*_1_ (second row) and *W*_0_ (third row) results in a decrease in the size of the Wnt domain. Results from the full 3D model after 50 min of simulation time (before the onset of migration).

We ran several simulations in which the aggregate parameters *F*_0_, *W*_0_, *F*_1_,*W*_1_ were varied by a factor of 10 up and down from their baseline value (represented with an asterisk, X*, see values in Table A in Supporting Information S6 Text. Parameter Estimation and Values) to study the effect on the resulting domain subdivision. Results after 50 min of simulation time (prior to migration) are shown in Fig 8. Trends match Fig 6, indicating that the analysis of the 1D model parallels the observations for the 3D model. The ligand profiles for both FGF and Wnt are also in good agreement with results from the 1D model.

### Migration

Having identified conditions for the formation of leading and trailing zones, we next asked what further conditions are required for collective migration of the PLLP. To probe this question, we assembled basic facts from the biology (surveyed above) supplemented, where needed, by reasonable minimal assumptions. In actual PLLP experiments, it is known that aside from the Wnt-FGF polarization of the tissue, the front and rear portions are also distinct in levels of chemokine receptors. Cells in the leading 60% of the PLLP express high levels of CXCR4b, whereas those in the remaining rear portion express high CXCR7b, [4]. This does not exactly coincide with the growth-factor zones: the leading 1/3 of the PLLP is the WntR activity zone [18]. However, here we made the simplification that from the standpoint of interactions with CXCL12a, the WntR-FGFR subdivision serves as reasonable proxy for the chemokine zone subdivision. One reason for this simplifying assumption is that we thereby avoid the further complications of untangling the signalling circuits that set up the zones of differential chemokine receptor expression. A second reason is that little is to be gained by this intermediate step currently, when the detailed biology of this step is largely unknown.

#### Basic migration model

**Fig 9:**
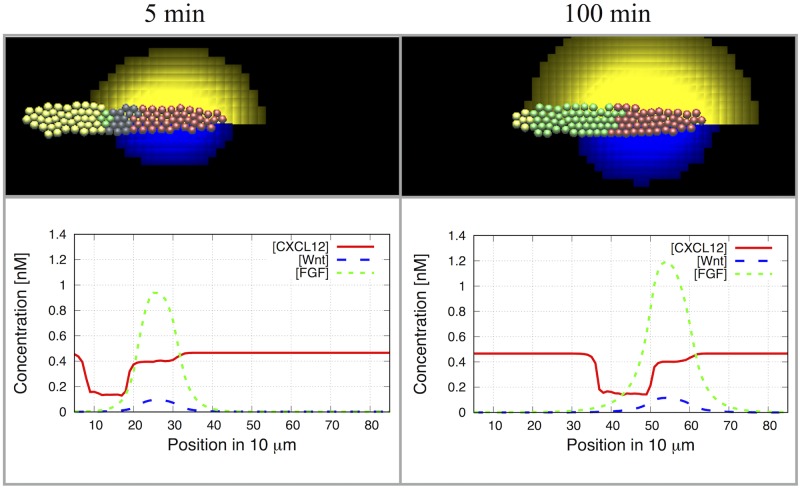
Successful migration in the discrete cell model. Simulations of the migrating primordium showing WntR expressing cells (red), FGFR expressing cells (green), WntR and FGFR expressing cells (yellow) and indeterminate cells (grey). Left: at 2 min, Right: at 100 min. Bottom row: concentrations of CXCL12a (red), FGF (green) and Wnt (blue). Parameters as in Table A in Supporting Information S6 Text. Parameter Estimation and Values. See Fig. C in Supporting Information S5 Text. Additional results and details, for a more detailed time sequence and Supporting Information S1 Movie. 3D simulation of PLLP migration, for a 4 hour movie of the simulation results.

To model the migration of the cells (Fig 9), we assumed a narrow long “stripe” of CXCL12a that was continually produced along the central long axis of the domain, as observed in experiments [5–7]. We further assumed the following: (a) Both leading and trailing cell types degrade CXCL12a, but trailing (FGFR expressing, green) cells do so at a 20-fold higher rate than leading (WntR expressing, red) cells ([10], and Fig. 1 in [19]). (b) Leading cells are assumed to migrate by chemotaxis up CXCL12a gradients. (c) FGFR expressing cells (shown in green) are pulled by their adhering neighbors and move by chemotaxis up FGF gradients. We consider alternative hypotheses in the Discussion.

Results of the basic simulation (Fig 9) are shown at two time points, in the very beginning of the simulation and 100 minutes later. (See Fig. C in Supporting Information S5 Text. Additional results and details, for a time-sequence and a movie in Supporting Information S1 Movie. 3D simulation of PLLP migration). The top panels show the evolution of the PLLP shape, the secretion of both Wnt and FGF ligands, the polarization, and the migration across the domain corresponding to about 250 *μ*m. The speed of motion is the same order of magnitude as the experimental observations. (1-2 *μ*m/min).

In the same figure, we also show the profiles of Wnt ligand (blue dashed line), FGF ligand (green dotted line), and CXCL12a (red solid line) across the lateral axis of the PLLP. It is evident that the Wnt and FGF ligand profiles form a sharp peak close to the front. We also observe a “trench” in the CXCL12a profile due to uptake and degradation of CXCL12a by the trailing cells. The simulated CXCL12a profile agrees well with results of [20].

#### Absence of FGF chemotaxis leads to migration failure

In our model, front cells migrating up CXCL12a gradients, and exert pulling forces on their neighbors, and cells further back actively migrate up a gradient of FGF ligand. We asked whether active migration of the rear is essential for collective migration, or whether the cells at the front might be “strong enough” to account for the PLLP motion.

We first estimated the viscous drag of a cell crawling through tissue. To do so, we used the fact that in this regime, cell speed is set by a balance between the active pulling forces exerted by a cell and the drag forces it experiences (*F* ≈ *μv*, where *μ* is a viscous drag coefficient). In cell collectives and epithelia, typical force generated by a cell has been measured to be on the order of a few nN [21, 22]. (This is reasonable, and represents roughly the combined effect of around 1000 actin filaments, each exerting a pushing force of about 1 pN at the cell edge [23].) The velocity of the cells in the PLLP is observed to be around 1 *μ*m per min [6]. This leads to an estimate of *μ* ≈ 6 ⋅ 10^−3^ dyne s/*μ*m for the viscous drag (1 dyne = 10^−5^ N) and is in the same ballpark as the estimate in [24] for a cell in a *Dictyostelium discoideum* slug. Cell motion is dominated by viscous drag, so inertial forces are insignificant and acceleration terms are absent in our model (contrasting with the model in [12]).

Using the above reasonable estimate of *μ*, we found that if only the cells at the front provide forward propulsion, then the PLLP hardly moves at all. Without an additional active propulsion mechanism for trailing cells, such as our FGF-directed chemotaxis, migration was either absent, or marginal at best. Furthermore, the PLLP became unrealistically elongated and the front occasionally tore apart from the rear (see Fig. A in Supporting Information S5 Text. Additional results and details). At the same time, if cells in the rear exert overly large motile forces relative to those at the front, then the PLLP shape becomes too wide and distorted, in disagreement with experimental observations. This points to the need for proper balance of the motile forces in the front and back of the PLLP.

### Cell growth and proliferation

It is well known that cell growth and division take place during the course of PLLP migration with the total number of cells doubling throughout the process [18, 25]. This fact led us to inquire how such cell proliferation contributes to the zone formation, migration, and overall shape of the PLLP. While it was originally thought that leading zone cells proliferate faster [18, 25], more recent experiments by two of us (AC and DDN) revealed a relatively constant and spatially uniform cell cycle length (539 ± 127 minutes) across the PLLP [26]. In the simulations the cell’s rate of growth in each time step is chosen from a uniform distribution between 0 and 0.001 min^−1^ (corresponding to a cell cycle between 0 and 1000 minutes). Cell division occurs when the volume of a given cell has doubled. The daughter cells have the same parameter values as the mother cell.

**Fig 10:**
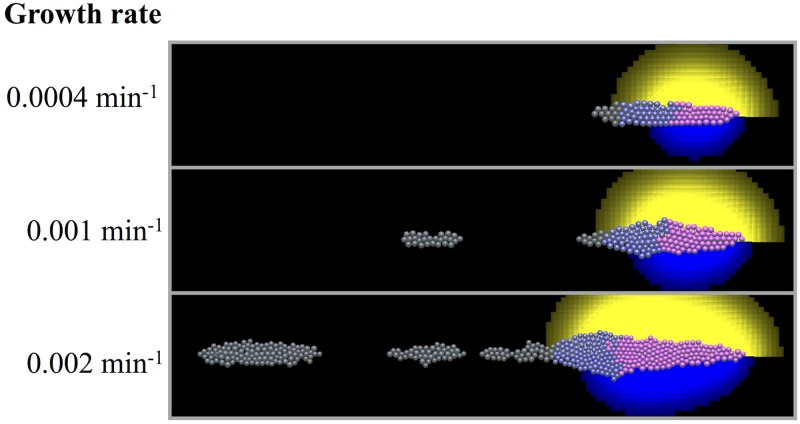
Effect of growth rate. Simulation results for three different growth parameters. Simulation time is 6 hours. As the growth rate is increased, the shape and size of the PLLP becomes distorted, and fails to match experimental observations.

Results with a range of growth rate values are shown in Fig 10. For the higher growth rate, we find that as the PLLP migrates and elongates, the cells at the back no longer detect FGF, and eventually break away from the rest of the PLLP.

### Comparison with published experiments

The spatiotemporal FGF and Wnt ligand distribution is hard to quantify experimentally. Hence, direct comparisons of model predictions to experimental observations are not possible for the chemical distributions of FGF and Wnt ligands. For this reason, we sought simple experimental comparisons that could (in)validate the computational model. We consequently compared our model behaviour to the results of laser ablation (cutting) experiments, even though such experiments have already been successfully recapitulated in a computational model [8]. We further simulated several mutants to check whether our predictions agree with what has been observed. In the experiments, staining has been done for Wnt signalling cells, using *lef1* as a marker, and for FGF signalling cells, using *pea3* as a marker. Hence we color the cells in our simulations based on signalling cell behaviour.

#### Laser ablation in silico

**Fig 11:**
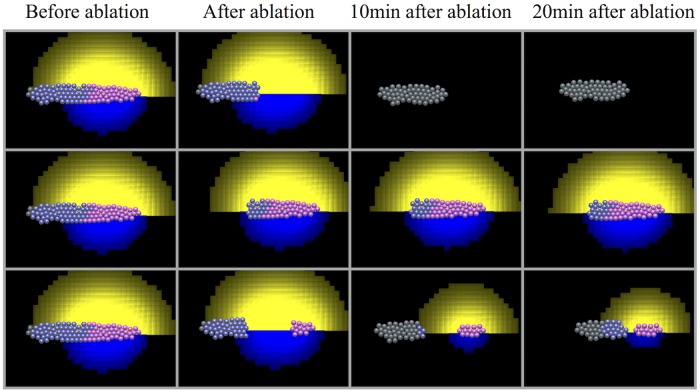
Recovery after partial ablation. As in laser ablation from [8], part of the PLLP is removed in the simulation, and recovery observed. Top row: the front segment (WntR active cells) is removed, leaving only FGFR active cells; the PLLP stalls and cannot continue. Middle row: the rear portion is removed, leaving a few FGFR cells behind; the PLLP migration continues. Bottom row: the middle segment is removed. Motion is stalled while the trailing FGFR active cluster catches up with the front. Once the clusters have merged, migration resumes.

To compare our model to results of [8], we performed ablation (cutting) experiments in which part of the simulated PLLP was removed. Results are shown in Fig 11. The left-most column shows the control PLLP before ablation, the second column shows the configuration at 12 min, directly after ablation, and the next two columns show what happens to the PLLP as a result.

We performed several such experiments. Following the establishment of clearly defined leading (pink) and trailing (purple) zones of Wnt and FGF activity, respectively, removal of the leading Wnt active zone (Fig 11, Row 1), initially leaves behind a PLLP entirely composed of FGFR active cells. However, in the absence of fresh FGF or Wnt signals produced by leading cells the entire remaining PLLP assumes an undefined state (gray) characterized by lack of both WntR and FGFR activity. Unsurprisingly, the same behaviour occurs when we remove both front and back (see Fig. B top panel in Supporting Information S5 Text). Additional results and details. Removing the trailing cells but leaving the middle fragment which contains FGFR active cells (Fig 11, Row 2), enables the PLLP to continue migrating. This result is comparable to the experiments where staining shows that the middle fragment has FGFR activity and the PLLP migration stalls temporarily and then starts up again. If we remove a larger part of the trailing PLLP, leaving no FGFR active cells behind the PLLP has no net forward movement, see Fig. B bottom panel in Supporting Information S5 Text. Additional results and details. About the parameter *W*_1_ we assumed the following: Initially, *W*_1_ is graded across the primordium, but when cells start dividing, daughter cells inherit the value of *W*_1_ from their mother cell.

When the middle portion of the PLLP is removed (Fig 11, Row 3), the back catches up to the front by chemotaxis towards the FGF gradient created by leading cells. The front and rear clusters merge, creating a gradient of CXCL12a and migration continues. These predictions are in agreement with both experiment and computational model in [8].

#### Comparison with mutants and other experiments

We compared the results of our simulations with experiments described in [4]. They used heat shock to overexpress the Dkk1 inhibitor and found decreased expression of FGF3 and FGF10 ligand. Staining shows no FGF activity (using *pea3* as a marker) and no Wnt activity (using *sef* as a marker). Inhibiting Dkk1 experimentally is comparable to decreasing *F*_0_ in our simulations, the IC_50_ parameter for Wnt inhibition. When *F*_0_ is lowered, fewer bound FGF receptors (and hence also less FGF ligand) is needed to inhibit Wnt receptor presentation Eq (4c). The model results (Fig 8) show that decreasing *F*_0_ 10 fold from the baseline level suppresses Wnt and FGF activity: there are no WntR active (pink) cells and no FGF ligand (yellow cloud) as a result.

SU5402 reduces the ability of the FGF receptor to respond to FGF ligand. According to experiments in [4], after treatment with the SU5402 inhibitor, WntR signalling activity takes over the entire primordium. Furthermore, using the SU5402 inhibitor just prior to migration leads to stalling of the primordium. In our model, this treatment corresponds to decreasing the steady state level of FGF receptors, *F*_1_. As shown in the third row of Fig 8, decreasing *F*_1_ increases the WntR activity domain and stalls migration, in agreement with experiments in [4].

Wnt ligand interacts with its receptor and inhibits APC-dependent β-catenin degradation to promote Wnt/β-catenin signalling. The *apc^mcr^* mutation reduces degradation, stabilizes β-catenin and promotes Wnt/β-catenin signalling independent of the Wnt ligand. As a result, FGF signalling is unable to inhibit Wnt signalling by promoting expression of a diffusible inhibitor of Wnt, Dkk1. Unregulated Wnt signalling in *apc^mcr^* mutants promotes high levels of Wnt-dependent FGF expression in the PLLP. Wnt signalling is unable to inhibit the FGF signalling that results from this unusually high levels of FGF expression, and the PLLP is left in a state characterized by high levels of both Wnt and FGF signalling, where mechanisms that normally determine FGF-Wnt mutual inhibition are lost. To reproduce these results in our simulations, we turned off FGF-Wnt mutual inhibition, which decouples the biochemistry of neighboring cells. Thus, as the 1D model predicts, all the cells express high levels of FGFR and WntR activities.

**Fig 12:**
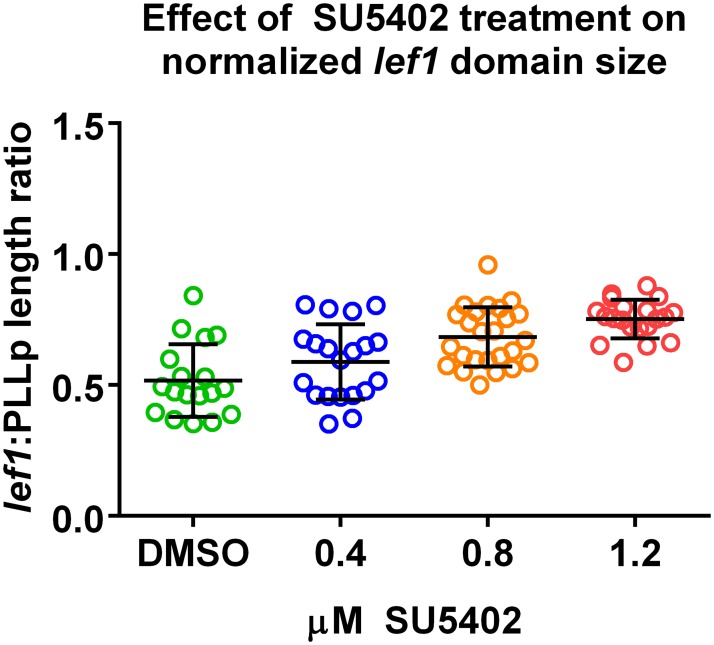
Dose response experiment using SU5402. The FGF receptor blocker SU5402 increases the size of the WntR activity domain in a dose-dependent manner (*lef1* used as a marker). The size of the *lef1* domain has been normalized relative to the size of the whole PLLP. A Linear regression analysis resulted in *y* = 0.2005*x* + 0.515. A one-way ANOVA reveals that the slope is significantly non-zero (0.2005 ± 0.03012). The 95% confidence interval for the slope is 0.1405—0.2605, with *R*^2^ = 0.3536, and *P* < 0.0001. These results validate the findings of our model.

In addition to these previously published results, we conducted new dose-response experiments to compare to simulation results of Figs 6 and 8, as described in Materials and Methods. Using the FGF receptor blocker SU5402 we show that the size of the WntR activity domain increases with increasing amount of the inhibitor, Fig 12. These results validate the predictions of our model where decreasing the parameter *F*_1_ (the steady state level of FGF receptors in the absence of inhibition) increases the size of the WntR activity zone.

## Discussion

Here, we investigated the lateral line primordium development and migration in zebrafish. In this intriguing system, a cluster of 100-200 cells maintains cohesion and directionality while migrating over a distance one order of magnitude longer than the cluster length. We first showed that the mutual inhibition of Wnt and FGF signalling can, on its own, set up the initial polarization of the PLLP into distinct leading and trailing domains under appropriate assumptions. Our model links ligand-receptor binding (in the Michaelis-Menten regime) to the inhibitory effect (of bound receptors on the antagonist receptors). Thereby, we could predict how the boundary between Wnt and FGF domains depends on parameters such as steady state receptor levels (in absence of inhibition) and sensitivity to inhibition by the antagonist bound receptors.

The theoretical model discussed here has more universal applicability, and variations on this theme could be at play in other differentiation processes of tissue subdivision. In particular, “crossed gradients” (i.e. gradients of opposite slopes) in the two ratios of receptor-ligand properties that we identified as *ϕ* and *ω* could produce a tissue with a central zone of overlapped influence (transition across the bottom panels of Fig 4), or with a sharp boundary (transition across the top panels of this figure). For our model of the PLLP specifically, such crossed gradients do not exist because the spatial variation of *ϕ* and *ω* stem from the FGF and Wnt ligand distributions, which are both graded in the same direction (increasing towards the front). Nevertheless, tissue subdivision occurs, once the PLLP evolves into a bistable state, with WntR activity locked into the front, and FGFR activity overtaking WntR in the back.

We have shown that the CXCL12a-expressing adhesive stripe provides directionality, as well as, coupled to active degradation by trailing cells, forms the guidance cue that directs PLLP migration along the lateral axis of the embryo. Given the viscous drag, it appears highly probable that all cells in the PLLP are actively motile. Simply having a “front engine” (a few cells at the front) pulling the entire mass would lead to migration stalling, and even fragmentation of the group, which is not observed under normal conditions. Chemotaxis is the simplest and most plausible mechanism for the active motion of trailing cells, though we do not excluded the possibility that trailing cells also actively follow leader cells through some other mechanism.

The shape and speed of the collective migrating mass depends on numerous factors such as rate of cell growth (which determines cell division rate), active crawling and adhesion of cells to one another and to the underlaying adhesive substrate. When these factors are increased or decreased beyond certain ranges, shape and speed are abnormal, recapitulating a variety of mutants. Wnt signalling regulates cell growth rate, and FGF signalling is implicated in cohesion. Both signalling types determine differentiation and the distinct roles of leading and trailing cells. WntR active front cells sense the CXCL12a gradient and steer the cluster. Rear cells eventually express CXCR7b chemokine receptors and degrade CXCL12a. The model demonstrates that this could form the self-propagating gradient to which WntR cells orient. FGFR active cells eventually engender the initiation of neuromast deposition that we have not yet addressed in this paper.

It is interesting to compare our results with those of previous modelling papers [8–12]. In Streichan et al. [9], the PLLP is modelled as a 1D rod, moving through a self-generated gradient of a single chemokine (CXCL12a), similar in flavor to “treadmilling” solutions in [27]. The rod binds the diffusible chemokine and moves with velocity assumed to be proportional to the gradient so created. The model for chemokine profile and rod position sustains a travelling wave solution, representing the steady-state long-range PLLP migration. The authors further considered deposition, assuming that below a threshold gradient of CXCL12a, the rear cells in a discrete 1D elastic array fall off. They derive an analytic expression for the migration velocity as a function of rates of ligand degradation and diffusion, the size of the PLLP and the rate of chemokine uptake. This model provides elegant analytic insights into minimal requirements for “self-generated” gradient chemotaxis in a general setting, while simplifying the biology and neglecting key features of the PLLP. For example, the authors do not consider interactions between CXCR7b and CXCL12a ligand, which is now well established in the literature. In comparison, our model begins to address this limitations by including some of these biochemical interaction, consequently limiting many of our results to numerical experiments.

Two of us (AC, DDN) previously crafted a Netlogo model [8, 10] as an aide to deciphering laser ablation experimental observations. The simulation uses “turtles” attached to one another with springs: when one moves, its neighbor moves with a slight lag, disallowing overlaps. This simulation had the advantage of close integration with experiments. While remaining a fairly elementary, it was able to capture key aspects of the biology, and explain experiments. A limitation was the absence of realistic forces of cell-cell interaction and friction. Consequently, a conclusion, based partly on the assumption of pulling exerted by few leading cells, was that cells respond to the CXCL12a concentration rather than CXCL12a gradient. In our simulation here, we endow a larger number of cells with the ability to sense, orient to, and migrate up the CXCL12a gradient to correct that feature. Furthermore, as we have argued, drag forces would stall the PLLP were it not for additional chemotaxis motion of the trailing cells.

Allena and Maini [11] described chemical signalling coupled to cellular mechanics in the PLLP using a reaction-diffusion finite element model. Cells are depicted by 2D Maxwell viscoelastic elements inside an elliptical PLLP. The incorporation of additional signalling (FGF and Wnt ligands, CXCR4b and CXCR7b chemokines as “readouts” of the ligand distribution), coupling mechanics and biochemistry, and the successful recapitulation of mutant behaviours are all strengths of this model. As in Allena and Maini [11], we have adopted a hybrid model to describe the PLLP dynamics in 3D. Our discrete cell model builds on limitations of [11] by including interactions between the PLLP and CXCL12a, relative motion and rearrangement of cells, and more direct representation of intercellular forces. In contrast to [11], our model also explains the initial subdivision of the PLLP into leading and trailing zones.

Another hybrid model [12] is based on cell-cell interaction, cell-substrate adhesion and cell differentiation. The model consists of discrete cells in 2D with a non-local sensing radius. The PLLP is described as a “flock” of cells accelerating via haptotaxis to CXCL12a, forces of Cucker-Smale alignment, attraction-repulsion, and chemotaxis to FGF gradient. The model captures the migration of the PLLP resulting from a graded distribution of CXCL12a. One issue with the representation of the PLLP as a “flock” is that macroscopic flocks and swarms generally operate in a high Reynold’s number regime, whereas we have chosen to neglect inertial forces and acceleration terms and to consider a low Reynold’s number regime for our cellular milieu. One interesting feature of [12] is that, by including cell-cell lateral inhibition in the trailing domain (and foci of FGF), their model can depict the formation of stable rosette structures within the PLLP (see their Figs 9 and 10). In contrast, we do not yet consider the rosette formation in our model but we base our cell motion and interactions on a closer concordance with recent biological results. In particular, we include downstream effects of Wnt/FGF signalling, i.e., formation of receptor activity domains, chemotaxis of trailing cells to FGF, and degradation of SDF1a (CXCL12a) in the trailing domain.

All models described above, including our own, would benefit from further experiments that could help to probe hypotheses or compare with predictions, in particular, the following experiments would be of primary interest:

- Implant additional PLLP cells into the primordium before migration begins. How would this affect the size of the Wnt domain and the migration? In our model, we assume that all WntR active cells are a source of Wnt ligand. This assumption will contribute to the size of the signalling domains. In the biological system, manipulating the size of the PLLP could validate the role of the Wnt10a ligand, or determine if the Wnt/β-catenin pathway remains active at the front of the PLLP.
- Similarly, we propose the inclusion of an external source of Wnt ligand (such as a Wnt-ligand containing bead) in an experiment with the wild-type primordium. Would this affect the size of the Wnt and FGF signalling zones? Can an external Wnt source rescue the signalling domain and migration in the migration-stalled *lef1*-mutants?
- Repeat the laser ablation experiment in a PLLP mutant with no cellular proliferation. Does the removal of the back segment still lead to PLLP migration or does it stall? In the previous experiments and our model, migration is rescued by the reformation of signalling domains as proliferation sufficiently lengthens the PLLP for the signalling domains to form. This could aid in the understanding of the role that proliferation and subsequent rosette organization play in maintaining PLLP migration.

Primarily, our suggested experiments seek to determine the roles of the Wnt and FGF ligands in sustained organization and migration of the PLLP. Does an initial signalling event start the PLLP migratory process, or, as we have assumed in our model, is a continuous source of Wnt ligand required to maintain the organization and migration of the PLLP?

In summary, we are not the first to model the PLLP migration, though ours is the first fully 3D simulation of its kind, with realistic cell-cell and cell-substrate forces. While the model is a drastic simplification of the biology, it nevertheless provides insights about the Wnt-FGF mutual inhibition, forces of propulsion and drag, and accounts for mutants. There are several opportunities for future work. First, there is the process of neuromast deposition that we consider in a future study. Second, there are many additional players in the signalling network responsible for segmenting the PLLP into leading and trailing zones, such as Dkk1 and *sef*, and including these players could aid in the understanding of their specific roles within the PLLP.

## Materials and methods

### 1D model simulations

In these simulations we treat the system in one spatial dimension across the long axis of the PLLP. We then define the spatial variable *x* with values in 0 ≤ *x* ≤ *L* where *x* = *L* is the front of the primordium and *x* = 0 is the back. All boundaries were modelled as impermeable (no flux). We used the MATLAB *pdepe* function to approximate solutions to the system. The sharp boundary between the leading and trailing domains makes numerical approximation difficult. For this reason, we only approximate the solution over a short time interval.

### Numerical determination of zone boundary position

To obtain the position of the boundary between signalling domains in Fig 6, the 1D Wnt-FGF model was simulated to steady-state with 20 distinct parameter values (*F*_0_, *F*_1_, *W*_0_, *p*_*F*_, *p*_*W*_, *b*), no flux boundary conditions, and initial conditions *W*(*x*, 0) = 0.01, *F*(*x*, 0) = 0.005, *W*_*R*_(*x*, 0) = 0.1, *F*_*R*_(*x*, 0) = 0.01. From the steady-state concentration profiles, the boundary between the signalling domains was identified as the intersection between the Wnt (*W*_*R*_) and FGF receptor (*F*_*R*_) curves. For Fig 6, we plotted the numerically calculated boundary position versus *p*/*p*_baseline_, the value of a given parameter, scaled by its baseline value in Table A in Supporting Information S6 Text. Parameter Estimation and Values. For the parameter sweeps, the following ranges were used: *b* ∈ [0, 0.05], *F*_0_ ∈ [0, 0.2], *F*_1_ ∈ [0, 2], *W*_0_ ∈ [0.0005, 0.0015], *p*_*F*_ ∈ [15, 35], *p*_*W*_ ∈ [2, 6].

### 3D discrete cell modelling framework

The discrete-cell computational framework is modified from software developed by one of us (EP) for signalling and motility in multicellular systems [24]. (See Supporting Information S4 Text. Discrete cell model and [24, 28] for technical details.) Cells are modelled as deformable ellipsoids in a three-dimensional (3D) domain. Cells move relative to one another, exert and respond to forces (chemotactic, adhesive, contact/exclusion, and random) in a low Reynolds number regime (neglecting inertia). FGF and Wnt ligand concentrations evolve in 3D based on a set of PDEs modified from Eqs. (19) in Supporting Information S4 Text. Discrete cell model to account for sources and sinks at individual cells. The ligands are depleted both through binding and internalization of the bound receptors as well as through nonspecific degradation in the domain.

Cells can orient and move in the direction of a chemical gradient. In addition to chemotactic forces, the cells also adhere to other cells and to the surface on which they crawl. The strength of the cell-cell adhesion force is the same for all types of cells. However, cells adhere more strongly to the CXCL12a expressing surface, and, since CXCL12a forms a narrow stripe, this accounts for the elongated shape of the primordium. While direct data is not available, it is likely that some differences in adhesion exist between cell types. Here we arbitrarily assumed that WntR expressing cells have a 2-fold higher adhesion to the CXCL12a stripe than do other types of cells. This does not have a major qualitative impact on our results. The net force acting on each cell is calculated and the cells are moved according to the equation of motion (25).

In a given simulation, the spatial concentration of the signalling molecules is calculated and updated, and then the cell positions are recomputed and updated each timestep.

### Dose response experiment

CldnB:lynGFP [6] embryos were treated from 32-36hpf with the indicated concentrations of SU5402 while shaking and then fixed in 4% PFA overnight. In situ hybridization for *lef1* transcript was performed as previously described [29]. Following in situ hybridization, embryos were stained with anti-GFP antibody to visualize the CldnB:lynGFP. The *lef1* ratio was computed by dividing the length of the *lef1* domain by the total length of the PLLP.

## Supporting information

S1 Text Model equations, derivation and scaling. (PDF)

S2 Text Formation of zones with graded initial Wnt receptor concentration. (PDF)

S3 Text Receptor positive feedback. (PDF)

S4 Text Discrete cell model. (PDF)

S5 Text Additional results and details. (PDF)

S6 Text Parameter estimation and values. (PDF)

S1 Movie 3D simulation of PLLP migration. (MPG)
