# Supplementary material for: Polarization and migration in the zebrafish posterior lateral line system
Source: PLoS Comput Biol. 2017 Apr 3;13(4):e1005451. doi: 10.1371/journal.pcbi.1005451 (PMC5393887; doi:10.1371/journal.pcbi.1005451)
Supplement: S1 Text — (PDF) [file pcbi.1005451.s001.pdf]

S1 Text. Model equations, derivation and scaling

The receptor equations, from Eqs. (4) are

$$\frac{dF_R}{dt} = E_F(W_B) - r_F F_R = \frac{E_{F,1}}{1 + (W_B/W_0)^n} - r_F F_R \tag{8a}$$

$$\frac{dW_R}{dt} = E_W(F_B) - r_W W_R = \frac{E_{W,1}}{1 + (F_B/F_0)^m} - r_W W_R. \tag{8b}$$

We wish to recast this in terms of F_R, W_R alone. Based on Michaelis-Menten kinetics, Eqs. (3), the bound receptors are given by

$$F_B(t) = \frac{F_R(t)F(t)}{K_F + F} = F_R(t)\frac{(F/K_F)}{1 + (F/K_F)} = F_R(t)s_F(F/K_F) \tag{9a}$$

$$W_B(t) = \frac{W_R(t)W(t)}{K_W + W} = W_R(t)\frac{(W/K_W)}{1 + (W/K_W)} = W_R(t)s_W(W/K_W) \tag{9b}$$

where we have defined the two functions

$$s_F(x) = \frac{x}{1+x}, \quad s_W(x) = \frac{x}{1+x}.$$

Now the ligand dependence is in the expressions s_F, s_W which represent the fraction of bound receptors for a given level of ligand concentration (relative to the ligand K_D). Note that both functions satisfy $0 \le s_j(x) \le 1$. We scale the FGF (respectively Wnt) ligand concentration by K_F (respectively K_W).

Substituting these expressions into the receptor dynamics equations leads to

$$\frac{dF_R}{dt} = r_F\left[\left(\frac{F_1}{1 + (W_R s_W/W_0)^m}\right) - F_R\right] \tag{10a}$$

$$\frac{dW_R}{dt} = r_W\left[\frac{W_1}{1 + (F_R s_F/F_0)^n} - W_R\right]. \tag{10b}$$

We scale the FGF receptors by F_1 and the Wnt receptors by W_1. Then the result is

$$\frac{dF_R}{dt} = r_F\left[\frac{1}{1 + (W_R/\omega)^m} - F_R\right] \tag{11a}$$

$$\frac{dW_R}{dt} = r_W\left[\frac{1}{1 + (F_R/\phi)^n} - W_R\right] \tag{11b}$$

where

$$\phi = F_0/(F_1 s_F), \quad \omega = W_0/(W_1 s_W). \tag{11c}$$

Note that as ligand concentration F (respectively W) decreases, s_F (respectively s_W) decreases, and so ϕ (respectively ω) increases. Now Eqs. (11)a,b are in the form of a mutual inhibition module and have a number of possible behaviours that depend on parameters ϕ and ω, as shown in Fig. 4.

In 1D, the ligand equations are

$$\frac{\partial F}{\partial t} = D_F \frac{\partial^2 F}{\partial x^2} - \delta_F F - \text{rate of binding} + \text{rate of production} \tag{12a}$$

$$\frac{\partial W}{\partial t} = D_W \frac{\partial^2 W}{\partial x^2} - \delta_W W - \text{rate of binding} + \text{rate of production} \tag{12b}$$

Ligand can bind to unoccupied receptors. Then, based on mass-action, the rates of binding are

$$\text{rate of binding to FGF receptors} = k_{F,\text{on}} F(F_R - F_B) = k_{F,\text{on}} F F_R (1 - \frac{F}{K_F + F}) \tag{12c}$$

$$\text{rate of binding to Wnt receptors} = k_{W,\text{on}} W(W_R - W_B) = k_{W,\text{on}} W W_R (1 - \frac{W}{K_W + W}) \tag{12d}$$

where the constants K_F, K_W are given by

$$K_F = \frac{k_{F,\text{off}} + k_{F,2}}{k_{F,\text{on}}}, \quad K_W = \frac{k_{W,\text{off}} + k_{W,2}}{k_{W,\text{on}}}. \tag{12e}$$

See Fig. 2 for these rate parameters. The binding rates can be simplified to

$$k_{F,\text{on}} F F_R (1 - \frac{F}{K_F + F}) = k_{F,\text{on}} F F_R \frac{K_F}{K_F + F} = k_{F,\text{on}} F F_R \frac{1}{1 + (F/K_F)}, \tag{12f}$$

$$k_{W,\text{on}} W W_R (1 - \frac{W}{K_W + W}) = k_{W,\text{on}} W W_R \frac{K_W}{K_W + W} = k_{W,\text{on}} W W_R \frac{1}{1 + (W/K_W)}. \tag{12g}$$

Similarly, the rates of production are

$$\text{rate of production of FGF ligand} = p_F W_B = p_F W_R \frac{W}{K_W + W} \tag{13}$$

$$\text{rate of production of Wnt ligand} = p_W W_B = p_W W_R \frac{W}{K_W + W}. \tag{14}$$

The reaction-diffusion PDEs for ligand concentrations can be written in the form

$$\frac{\partial F}{\partial t} = D_F \frac{\partial^2 F}{\partial x^2} - \delta_F F - k_{F,\text{on}} F F_R \frac{1}{1 + (F/K_F)} + p_F W_R \frac{W}{K_W + W} \tag{15a}$$

$$\frac{\partial W}{\partial t} = D_W \frac{\partial^2 W}{\partial x^2} - \delta_W W - k_{W,\text{on}} W W_R \frac{1}{1 + (W/K_W)} + p_W W_R \frac{W}{K_W + W}. \tag{15b}$$

We now put these in dimensionless form by the following scaling:

$$F = F^* \bar{F}, \quad W = W^* \bar{W},$$

where *s are dimensionless and bars are the scales. We choose $\bar{F} = K_F, \bar{W} = K_W$ as noted earlier. We also have scales for distance ($x = x^* L$) and for the receptor levels ($F_R = F_R^* F_1, W_R = W_R^* W_1$) as discussed previously. This gives the dimensionless (except for time) model:

$$\frac{\partial F}{\partial t} = \mathcal{D}_F \frac{\partial^2 F}{\partial x^2} - \delta_F F - \kappa_F F_R \frac{F}{1 + F} + \rho_F W_R \frac{W}{1 + W} \tag{16a}$$

$$\frac{\partial W}{\partial t} = \mathcal{D}_W \frac{\partial^2 W}{\partial x^2} - \delta_W W - \kappa_W W_R \frac{W}{1 + W} + \rho_W W_R \frac{W}{1 + W} \tag{16b}$$

where

$$\mathcal{D}_i = \frac{D_i}{L^2}, \quad \kappa_F = k_{F,\text{on}} F_1, \quad \kappa_W = k_{W,\text{on}} W_1, \quad \rho_F = \frac{p_F W_1}{K_F}, \quad \text{and} \quad \rho_W = \frac{p_W W_1}{K_W}. \tag{16c}$$
