# Supplementary material for: Polarization and migration in the zebrafish posterior lateral line system
Source: PLoS Comput Biol. 2017 Apr 3;13(4):e1005451. doi: 10.1371/journal.pcbi.1005451 (PMC5393887; doi:10.1371/journal.pcbi.1005451)
Supplement: S2 Text — (PDF) [file pcbi.1005451.s002.pdf]

S2 Text. Formation of zones with graded initial Wnt Receptor concentration

Instead of grading the steady-state levels of Wnt Receptors, W_1, across the PLLP to form signalling domains, it is also possible to form signalling domains by imposing an initial gradient on W_R (with constant W_1). In this case, we assume that the initial concentration of Wnt receptors, $W_R(x, 0)$, has a steeper gradient as the parameter W_1 above. That is, we set

$$W_R(x, 0) = 0.1x + 0.03, \quad \text{and} \quad W_1(x) = W_1 = 0.03. \tag{17}$$

Results of the 1D spatial simulation are shown in Fig. A and Fig. B. Signalling domains form with the same parameters as before (Table A in S6 Text. Parameter Estimation and Values) except with $p_F = 15$, $p_W = 5$, and $b = 0.1$ (b is the slope of the gradient). In addition, initial conditions are $W(x, 0) = 1$, $F(x, 0) = 1$, and $F_R(x, 0) = 0.35$. This starts the Wnt-FGF signalling network in a bistable state ($\phi < 1, \omega < 1$). An initial gradient of W_R across the PLLP results in a different initial condition for each spatial location x. Thus, depending on which basin of attraction the initial condition is in at each x, the system will either to a Fgf or Wnt signalling state. Subsequent ligand production and secretion ensures that the signalling domains are formed by ensuring that the Wnt-FGF signalling phase-space does not further evolve over time (ϕ, ω remain below 1).

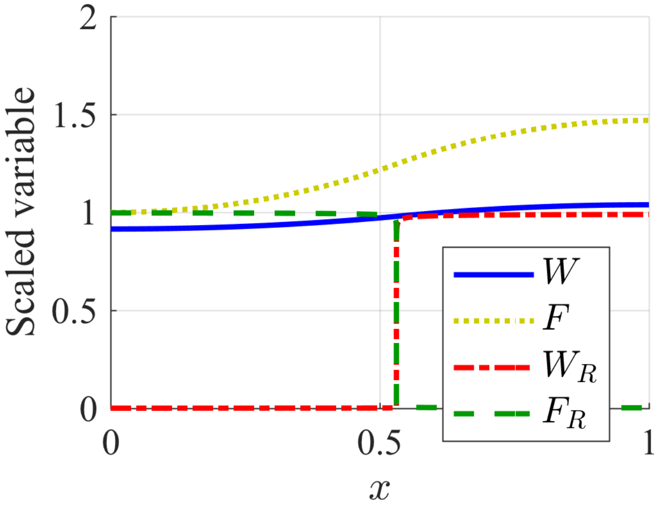

Figure A. A gradient in initial Wnt receptor concentration can induce signalling domains in the PLLP. The concentration profiles of the ligand and receptor activity are plotted at $t = 5$ minutes (after the signalling domains have formed), which reveals the formation of a sharp boundary between the leading and trailing zones.

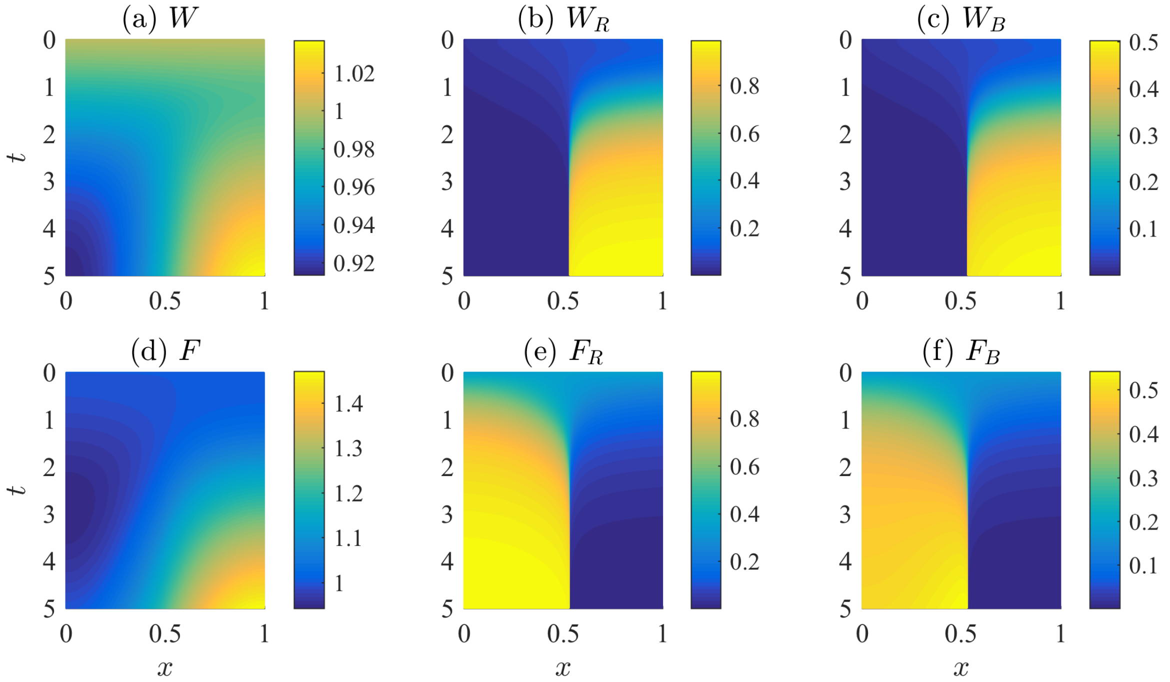

Figure B. A gradient in initial Wnt receptor concentration can induce signalling domains in the PLLP. Kymographs of the concentration profiles of the ligand and receptor activity are plotted to reveal the formation of the leading and trailing zones.
