# Supplementary material for: Polarization and migration in the zebrafish posterior lateral line system
Source: PLoS Comput Biol. 2017 Apr 3;13(4):e1005451. doi: 10.1371/journal.pcbi.1005451 (PMC5393887; doi:10.1371/journal.pcbi.1005451)
Supplement: S3 Text — (PDF) [file pcbi.1005451.s003.pdf]

S3 Text. Receptor positive feedback

We also considered a variant of the model that allows for positive feedback between each signalling type and its own receptor presentation rate.

Briefly, after similar scaling, such a model leads to modified equations of the form

$$\frac{dF_R}{dt} = r_F \left[\frac{1}{1 + (W_R/\omega)^m} \left(1 + \gamma \frac{F_R^p}{\phi_2^p + F_R^p} \right) - F_R \right] \tag{18a}$$

$$\frac{dW_R}{dt} = r_W \left[\frac{1}{1 + (F_R/\phi)^n} \left(1 + \alpha \frac{W_R^q}{\omega_2^q + W_R^q} \right) - W_R \right]. \tag{18b}$$

Here γ, α are positive feedback magnitudes for each of the FGFR and WntR signalling systems, and ϕ_2, ω_2 are the respective "EC_{50}" parameters for that feedback (i.e. concentrations at which the positive feedback is 50% of its maximal strength). The case $\gamma = \alpha = 0$ reduces to the previous model with mutual inhibition alone.

If the positive feedback terms are Michaelian ($p = q = 1$) terms, then the model is hardly changed qualitatively, although steady states are of somewhat greater magnitude. (This stems from the fact that the activation terms are multiplied by factors that approach $1 + \gamma$ and $1 + \alpha$ for large values of F_R, W_R.) Even in the case that $p, q \geq 2$, there are parameter regimes in which little qualitative change is noticeable, as shown in Fig. A. However, some exceptions are noteworthy, as illustrated in Fig. B.

Setting $W_R = 0$ in Eqn. (18a) leads to a well-known positive feedback ODE for F_R that is known to have a bistable regime for an appropriate range of parameters when $p \geq 2$. Similarly, setting $F_R = 0$ in Eqn. (18b), with $q \geq 2$ can produce a bistable W_R system. It comes as no surprise, therefore, that the system as a whole can have up to four stable steady states, with all possible combinations of low and high levels of F_R and W_R. Some extreme examples of possible outcomes are shown in Fig. B. Such behavior occurs when the Hill coefficients p, q are relatively large, e.g ≈ 10, so that positive feedback turns on very rapidly near its threshold. Experimentally, it is not clear whether cells in the PLLP exhibit such diversity of states.

It is not possible to solve for steady states of Eqs. (18) analytically in the general case, since the nonlinearities lead to polynomial equations. However, good insight can be obtained once more by approximating the rational functions by step functions. This "sharp switch approximation" leads to piecewise constant approximation of the nullclines, which are then easy to handle. The positive feedback terms turn on when $F_R = \phi_2$ and when $W_R = \omega_2$, respectively. It is then easy to show that bistability in either the F_R or the W_R signalling system takes place when $1 < \phi_2 < 1 + \gamma$ or $1 < \omega_2 \leq 1 + \alpha$,

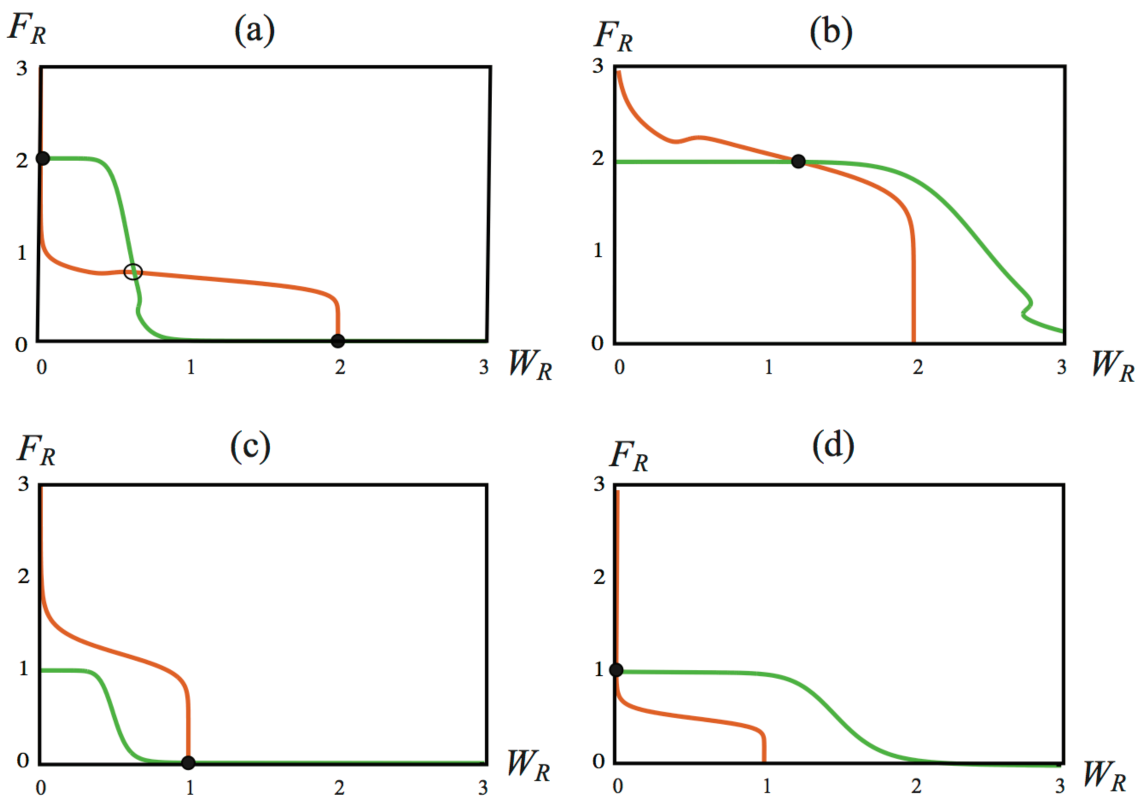

Figure A. Positive feedback with little effect. Introducing positive feedback into the model, as per Eqs. (18) has minor effect in some cases. Shown here are nullclines of the revised model in four cases that resemble the dynamics of the system with no positive feedback. Parameter values: (a) $\phi = 0.7, \omega = 0.6, \omega_2 = 0.5, \phi_2 = 0.4$, (b) $\phi = 2.1, \omega = 2.5, \omega_2 = 0.5, \phi_2 = 0.4$, (c) $\phi = 1.2, \omega = 0.5, \omega_2 = 2.5, \phi_2 = 2.4$, (d) $\phi = 0.5, \omega = 1.5, \omega_2 = 2.5, \phi_2 = 2.4$. In all cases, $n = m = p = q = 10, \gamma = 1.0, \alpha = 1.0$. Black dots are stable steady states and white dots are unstable.

respectively. Configurations shown in Fig. B are then possible. As an example, panel (d) is obtained when $\phi > 1 + \gamma > \phi_2 > 1$ and $\omega > 1 + \alpha > \omega_2 > 1$.

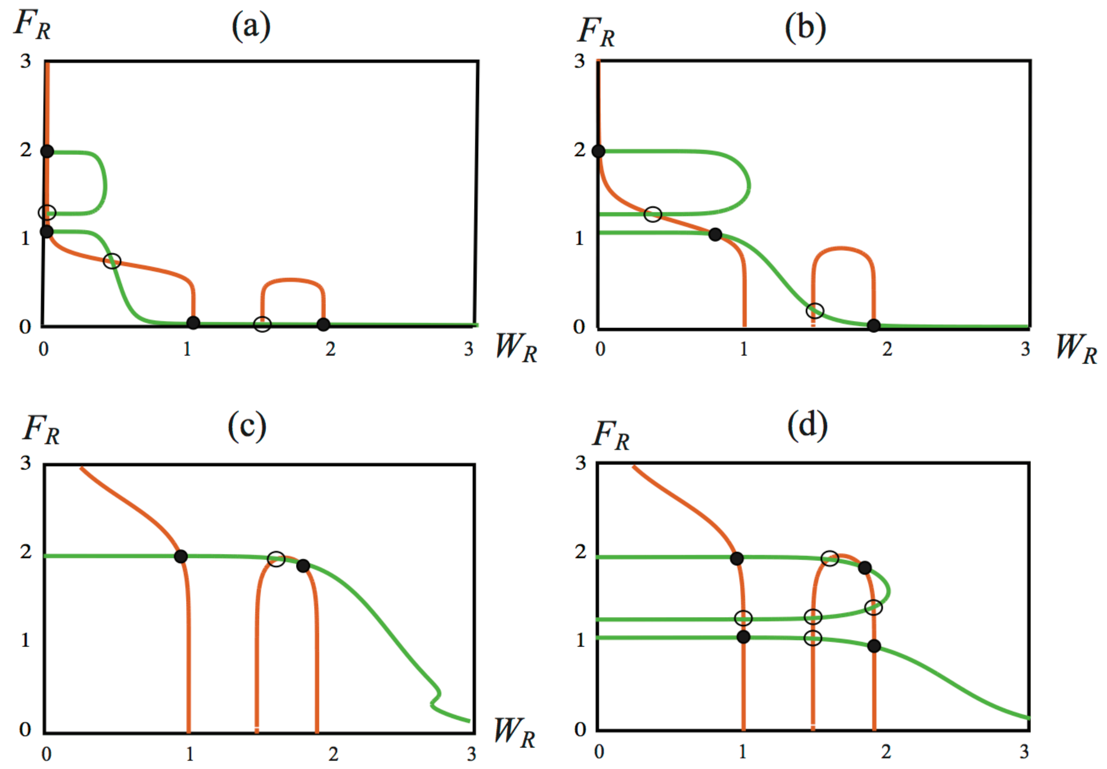

Figure B. Positive feedback can have major effect. Examples of possible $W_R F_R$ phase planes showing the effect of introducing positive feedback into the model, as per Eqs. (18). In these cases one or both of the Wnt or FGFare bistable, which introduces the possibility of multiple steady states. The curves on the plot are nullclines of the system of differential equations (18). Parameter values used were: (a) $\phi = 0.7, \omega = 0.5, \omega_2 = 1.5, \phi_2 = 1.4$, (b) $\phi = 1.2, \omega = 1.3, \omega_2 = 1.5, \phi_2 = 1.4$, (c)$\phi = 2.7, \omega = 2.5, \omega_2 = 1.5, \phi_2 = 0.4$, (d) $\phi = 2.7, \omega = 2.5, \omega_2 = 1.5, \phi_2 = 1.4$. In all cases, $n = m = p = q = 10, \gamma = 1.0, \alpha = 1.0$. Black dots are stable steady states and white dots are unstable.
