# Supplementary material for: Polarization and migration in the zebrafish posterior lateral line system
Source: PLoS Comput Biol. 2017 Apr 3;13(4):e1005451. doi: 10.1371/journal.pcbi.1005451 (PMC5393887; doi:10.1371/journal.pcbi.1005451)
Supplement: S4 Text — (PDF) [file pcbi.1005451.s004.pdf]

S4 Text. Discrete cell model

The discrete model is adapted from software (written in C) by Eirikur Palsson [24] with 3-D output images and movies generated by openGL. The cells are represented by deformable ellipsoids of finite volume with mechanochemical interactions. The time step is 0.01 min. Random and chemotactic active forces govern cell motion, attractive adhesive forces are associated with cell-cell and cell-substrate contact, and a short-range repulsive force prevents cells from occupying the same volume. Cell motion is calculated from net force acting on each cell.

Chemical concentration The surfaces of cells act as sources/sinks for the secreted ligands. To track the ligand concentration, we solve the following (unscaled) PDEs on a 3-D cartesian grid (with each grid cube representing 10^3 μm^3):

$$\frac{\partial F}{\partial t} = D_F \nabla^2 F - \delta_F F - \kappa_F F_R \Omega_F + p_F \Omega_W \tag{19a}$$

$$\frac{\partial W}{\partial t} = D_W \nabla^2 W - \delta_W W - \kappa_W W_R \Omega_W + p_W \Omega_W \tag{19b}$$

together with Eqn. (6). Cells are not point sources but instead can overlap several lattice cubes. The overlap depends on the location of the cell relative to the center of the grid lattice, i.e. the displacement (Δx,Δy,Δz), where i =int(x) and $\Delta x = i - x$. Similar for y and z. When the displacement is zero the cell is exactly in the center and overlaps no other lattice cube. The factors $\Omega_{W,F}$ represent the proportion of cell surface area in each lattice cube ($0 \leq \Omega_{W,F} \leq 1$). Ω_W represents the WntR expressing cells (shown in red) and Ω_F represents FGFR expressing and undetermined cells (green and grey). Eqs. (19) are discretized to the grid so that $\Omega_W(x, y, z) = \Omega_W^{ijk}$ where

$$\Omega_W^{ijk} = \sum_{q \in \mathcal{N}(i,j,k)(q)} \frac{S_{cell-q}^{ijk}}{S_{cell-q}}.$$

$\mathcal{N}(i, j, k)(p)$ stands for all the cells that overlap lattice cube i, j, k. S_{cell-q} is the total surface of cell q, and S_{cell-q}^{ijk} the fraction of cell-q's surface area in lattice cube ijk. In principle, the fraction of the cell surface area S_{cell-q}^{ijk} could be obtained from a precalculated "Surface area" lookup table for ellipsoids based on the displacement (Δx,Δy,Δz), created once at the beginning by numerical integration. We chose to simplify the process by approximating the ellipsoid by rectangular boxes, which makes it easy to calculate S_{cell-q}^{ijk} from the displacement (Δx,Δy,Δz). We verified that results based on this approximation did not differ greatly from the results of true ellipsoidal surface area look-up table.

The cells "sense" the local gradient concentration. As opposed to the 1D simulations, since cells are now discrete, we must account for the fact that each has a finite sensing size that possibly overlaps several voxels in the chemical grid. Hence, we take an average over those voxels, so that the average local concentration detected by a given cell is calculated using the following equation (where ijk refers to a given lattice cube):

$$[L]_{cell-q} = \sum_{i=l-1}^{i=l+1} \sum_{j=m-1}^{j=m+1} \sum_{k=n-1}^{k=n+1} \frac{S_{cell-q}^{ijk}}{S_{cell}} [L]^{ijk}. \tag{20}$$

Here L is the ligand (Wnt or FGF) and $[L]^{ijk}$ is the ligand concentration in lattice cube ijk.

We similarly correct for finite cell size by distributing the secreted ligands into surrounding voxels in proportion to the fraction of cell surface inside a given voxel.

$$L_{q-sec}^{ijk} = \sum_{q \in (ijk)} \frac{S_{cell-q}^{ijk}}{S_{cell}} p_L. \tag{21}$$

Here L_{q-sec}^{ijk} is the concentration of ligand secreted by cell q in lattice cube ijk and p_L is the total rate of ligand secreted by the cell (p_L takes on the values p_W and p_F, for Wnt and FGF ligands, respectively, see Table A in S6 Text. Parameter Estimation and Values).

Ligands are removed by cells (rate k_{loc}) and have some overall decay (k_{ext}). The rate of removal, corrected for finite cell sizes is taken to be

$$k_{deg}^{L^{ijk}} = k_{ext}^{ijk,L} + \sum_{q \in (ijk)} \frac{S_{cell-q}^{ijk}}{S_{cell}} k_{loc}^{L}. \tag{22}$$

Forces We summarize here the forces on and between cells and how these are computed.

The **active force**, $\mathbf{F}_{active}$ represents the traction force that a cell applies to the myoseptum. Here we assumed that active forces result from chemotaxis to FGF ligand and/or to CXCL12a, but other possibilities (such as adhesion gradients, gradients of substrates rigidity, or simple active propulsion) could be used in generalized variants of the model. In our discrete model, the active force has a fixed constant magnitude whenever the levels of FGF and CXCL12a are detectable (i.e. above some threshold). The direction of the active force is determined from a vector sum of the local gradients of FGF and CXCL12a. Failing to detect chemicals, the cell applies a weaker force (0.1nN) in a random direction. This allows the cell to deviate by up to 20° of its previous direction of motion.

The **myoseptum adhesion force** is only in the z direction, acting to attach cells to the 2D substrate; as this force is orthogonal to the xy plane, it has no effect on the migration of the PLLP, nor

on drag forces (which act to oppose that migration). A small additional force of adhesion to the CXCL12a stripe acts only in the y direction and locally pulls cells towards the stripe. The magnitude of this force is $< 10\%$ that of the adhesion to the myoseptum. The detailed dependence on cell type or cell state is unknown, but we arbitrarily assumed that WntR expressing cells have a 2-fold higher adhesion to CXCL12a than other cells, while having the same adhesion to the myoseptum. (We verified that this factor of 2 did not make a large qualitative difference in behavior.)

The **cell-cell interface forces** include both adhesion and volume-exclusion (attraction and repulsion). Since ellipsoids are not space-filling, whereas cells in the PLLP are a compact mass, we allow the ellipsoids to overlap somewhat at equilibrium. Further overlap is avoided by a power-law **exclusion force** that depends on x, the distance between the cell surfaces along the vector connecting their centers. The **adhesion force** between two cells acts along the same axis, with a magnitude that goes to zero for cells that overlap or that are far apart. This force pulls cells towards their equilibrium spacing, but acts locally. The net force at cell interfaces, $\mathbf{F}_{interface}$, is calculated as

$$\begin{aligned} \mathbf{F}_{interface} &= \begin{cases} -F_{adhesion}\chi\left[(x+x_0)e^{-\lambda(x+x_0)^2)} - v_0 e^{-\lambda x^2}\right]\cdot\frac{\vec{\mathbf{r}}_{ij}}{\|\vec{\mathbf{r}}_{ij}\|} & \text{if } x > 0, \\ F_{exclusion}\chi(-x)^{\frac{9}{5}}\cdot\frac{\vec{\mathbf{r}}_{ij}}{\|\vec{\mathbf{r}}_{ij}\|} & \text{if } x \le 0. \end{cases} \\ \chi &= \frac{r_{cell}}{2}\left(\frac{1}{d_i}+\frac{1}{d_j}\right), \quad x = \frac{d-d_{min}}{r_{cell}}. \end{aligned} \tag{23}$$

Here $\vec{\mathbf{r}}_{ij}$ is a vector between the centers of cells i and j, r_{cell} is the cell radius, d_i and d_j are the distances from the centre to the cell surface along the vector $\vec{\mathbf{r}}_{ij}$ for ellipsoid i and j, respectively, d is the distance between the surfaces of two neighboring ellipsoids along the vector $\vec{\mathbf{r}}_{ij}$ (i.e. $d = \|\vec{\mathbf{r}}_{ij}\| - d_i - d_j$) and d_{min} determines the point at which the cells are in the equilibrium state where the adhesion and exclusion terms balance (see Fig. 11 in [24]). In our simulations $d_{min} = -0.1r_{cell}$. This allows the ellipsoids to overlap and corrects for the fact that cells are not actually rigid ellipsoids. (It also allows neighboring cells to have a finite contact area, which would not be the case for impermeable rigid ellipoids.) The constants x_0 and v_0 are chosen to ensure that when cells are touching, i.e. when $x = 0$, both $F_{adhesive} = 0$ and $F'_{adhesive}(x) = 0$.

Cell motion The motion of each cell is determined by the net force $\mathbf{F}_{cell}$.

$$\mathbf{F}_{cell} = \mathbf{F}_{active} + \sum_{N}\mathbf{F}_{interface}, \tag{24}$$

The position of the cell is updated by the (low Reynold's number regime) equation of motion,

$$d\vec{x}/dt = \mathbf{F_{cell}}/\mu, \tag{25}$$

where μ is a drag coefficient.

Cell shape The cell is modelled as a viscoelastic ellipsoid with semi-minor axes a, b, c. Each axis contains a Maxwell element (spring with spring constant κ_1 in series with a dashpot whose frictional coefficient is μ_1) in parallel with a spring (with spring constant κ_2). (See [24] for typical mechanical parameters.) The forces acting on a cell are resolved onto the three ellipsoidal axes and the shape of the cell is obtained from these mechanical elements under volume conservation.

$$\frac{dr_i}{dt} = \frac{\kappa_1(F_i - F_{mod})}{\mu_1(\kappa_1 + \kappa_2)} + \frac{dF_i/dt}{(\kappa_1 + \kappa_2)} - r_i \frac{\kappa_1 \kappa_2}{\mu_1(\kappa_1 + \kappa_2)}, \tag{26}$$

$$r_a r_b r_c = (r_a + \Delta r_a)(r_b + \Delta r_b)(r_c + \Delta r_c) = V_{ellipse}/(\frac{4}{3}\pi), \tag{27}$$

where r_i is the length of either the a, b or c axis in units of 10 μm and F_{mod} is calculated from the volume constraint in each time step by solving Eqn. (26) to find Δr_a, Δr_b and Δr_c under the constraint of Eqn. (27). Allowing cells to deform is significant, as it is easier for deformable cells to move past neighbors in 3D systems.

Overall simulation protocol In all cases, we initiate the system with a rectangular configuration of 100 uncommitted (grey) cells, and no ligand of either type. We first simulate the system with cell-cell, cell-CXCL12a adhesion and cell contact forces, but no FGF nor Wnt ligand. This allows the transients due to initialization to be resolved. The time-stepping proceeds as follows:

1. The local concentration of the signalling molecules around each cell is calculated (Eqn. (20)).
2. The secreted ligands are distributed into the lattice cubes (Eqn. (21)) and the diffusion and depletion of FGF, Wnt and CXCL12a is calculated (Eqs. (19) and (6)).
3. Cells orient towards the FGF gradient (green, FGF expressing cells) or CXCL12a gradient (red, Wnt expressing cells) if ligand is at a detectable level. Otherwise, cells orient in a random direction, biased towards the previous direction of motion.
4. All the active and exclusive forces acting on each cell are calculated.

5. Cells are moved according to the equation of motion (Eqn. (25)).

6. This is repeated for every time step.

Table A. Summary of 3D Model Parameters.

Parameter	Definition	Units	Value
μ_{ECM}	Viscosity	dyne per s/mm	10^{-3}
r	Cell radius	μm	5
$F_{adhesion}$	Cell-Cell adhesion	nN	0.8
F_{active}	Force of chemotaxis	nN	0.7
$F_{exclusion}$	Exclusion force	nN	50
	Random force	nN	0.1
	Cell-surface adhesion	nN	1.1
D_C	CXCL12a diffusion	μm^2 min^{-1}	20
α	CXCL12a production	nM	0.2
δ_C	CXCL12a external decay	min^{-1}	0.2
k_{CW}	CXCL12a decay by WntR cells	min^{-1}	0.05
k_{CF}	CXCL12a decay by FGFR cells	min^{-1}	1.0
p_W	Wnt secretion	nM	3.0
p_F	FGF secretion	nM	0.5
	FGF external decay	min^{-1}	0.15
	Wnt external decay	min^{-1}	0.15
K_F	FGF M-M constant	nM	1.0
K_W	Wnt M-M constant	nM	1.0

Further details on this computational framework can be found in [24,28].
