# Supplementary material for: Polarization and migration in the zebrafish posterior lateral line system
Source: PLoS Comput Biol. 2017 Apr 3;13(4):e1005451. doi: 10.1371/journal.pcbi.1005451 (PMC5393887; doi:10.1371/journal.pcbi.1005451)
Supplement: S5 Text — (PDF) [file pcbi.1005451.s005.pdf]

S5 Text. Additional results and details

No FGF chemotaxis If trailing (FGF expressing) cells do not chemotax towards the FGF ligand, then the shape of the PLLP and its migration is no longer biological, as shown in Fig. A.

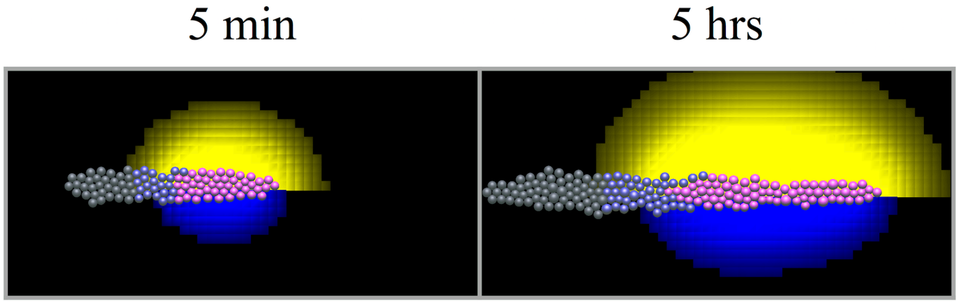

Figure A. Abolished FGF chemotaxis If FGF expressing cells in the trailing part of the PLLP do not chemotax towards FGF ligand, then the PLLP becomes elongated, and its shape no longer resembles the experimental observed PLLP shape.

Laser ablation In the top panel of Fig. B we show a simulation of the removal of both front and back of the PLLP. The remaining middle segment stalls. Removing a larger part of the back of the PLLP removes all of the FGFR active cells resulting in no net forward migration of the remaining PLLP.

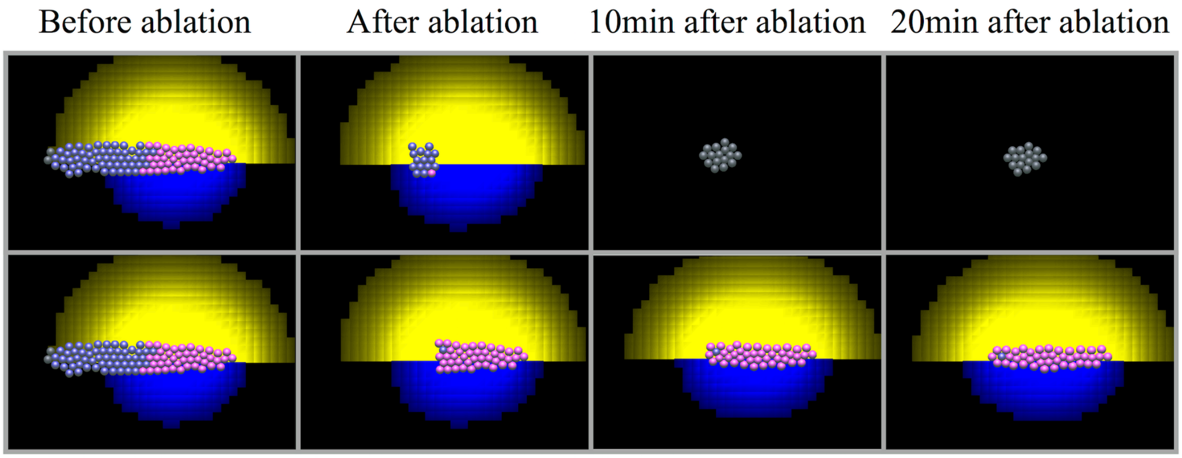

Figure B. Laser ablation experiment Top panel: Both the front and the back of the PLLP are removed resulting in the middle segment stalling. Bottom panel: A large part of the back is removed, leaving no FGFR active cells behind. The PLLP has no net forward movement.

Successful migration, details We show a more detailed time sequence of migration phases in Fig. C. See also Supporting Information S1 Movie. 3D simulation of PLLP migration, for a 4 hour simulation of the migrating PLLP.

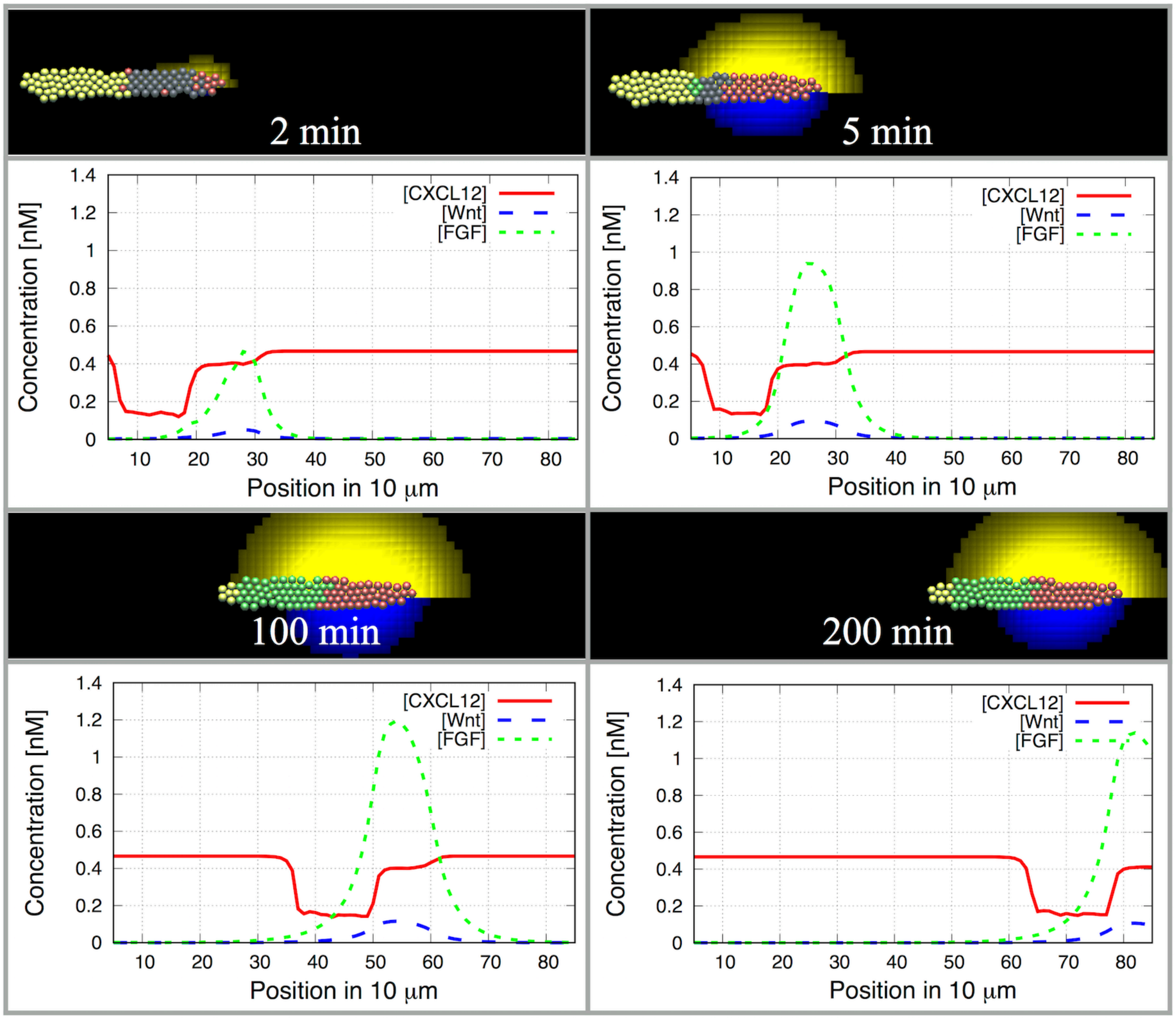

Figure C. Time sequence of migration of the primordium (As in Fig. 9 with four time points.) WntR (red) and FGFR (green) expressing cells, cells expressing both (yellow) and undetermined cells (grey). The concentrations of the three ligands, CXCL12a (red line), Wnt (blue long dashes) and FGF (green short dashes) are shown. Parameters as in Table A in S6 Text. Parameter Estimation and Values.
