# Supplementary material for: Polarization and migration in the zebrafish posterior lateral line system
Source: PLoS Comput Biol. 2017 Apr 3;13(4):e1005451. doi: 10.1371/journal.pcbi.1005451 (PMC5393887; doi:10.1371/journal.pcbi.1005451)
Supplement: S6 Text — (PDF) [file pcbi.1005451.s006.pdf]

S6 Text. Parameter Estimation and Values

We emphasize that our model is a qualitative, rather than quantitative predictive model since much of the biological detail, and most of the biological rate constants are unknown and/or not easily measurable. Nevertheless, we have attempted to base many of our parameter values on information about ligands and cells in the biological literature. As in many current computational models, these values come from diverse species and experimental conditions, and should be interpreted with caution.

Table A lists the parameters, their units and values.

Table A. Summary of Model Parameters.

Parameter	Definition	Units	Value	Reference
F_0	FGF IC50	nM	0.1	set
F_1	steady-state FGF receptor concentration	nM	1	set
W_0	Wnt IC50	nM	0.001	set
W_1	steady-state Wnt receptor concentration	nM	0.03	[30]
$n = m$	exponents	dimensionless	5	set
δ_F	FGF ligand decay rate	min^{-1}	0.002	[31]
δ_W	Wnt ligand decay rate	min^{-1}	0.03	[32]
r_F	FGF receptor endocytosis rate	min^{-1}	1	set
r_W	Wnt receptor endocytosis rate	min^{-1}	1	set
$k_{F,\text{on}}$	FGF ligand binding rate	$\text{nM}^{-1}\text{min}^{-1}$	0.1	[33]
$k_{W,\text{on}}$	Wnt ligand binding rate	$\text{nM}^{-1}\text{min}^{-1}$	0.005	[30]
D_F	FGF diffusion coefficient	$\mu\text{m}^2\ \text{min}^{-1}$	600	estimated
D_W	Wnt diffusion coefficient	$\mu\text{m}^2\ \text{min}^{-1}$	600	estimated
p_F	FGF secretion rate for W_1 gradient	min^{-1}	24	set
p_W	Wnt secretion rate for W_1 gradient	min^{-1}	4	set
b	W_1 gradient slope	nM μm^{-1}	0.01	set
p_F	FGF secretion rate for W_R gradient	min^{-1}	15	set
p_W	Wnt secretion rate for W_R gradient	min^{-1}	5	set
b	W_R gradient slope	nM μm^{-1}	0.1	set

Primordium size and units We use units of μm for length, and minutes for time. The PLLP is on the order of 100-200 μm long [8]. The entire primordium is estimated to contain about 125 cells at the onset of migration.

Ligand-receptor binding kinetics and typical concentrations To begin, we make the assumption that the endocytosis rates of Wnt and FGF receptors are the same: $r_W = r_F$. The receptor endocytosis rates appear in the scaled receptor ODE equations as a time-scaling factor only, and given the lack of information on endocytosis rates, we set $r_W = r_F = 1$. We set $n = m = 5$, but values $n \neq m \geq 2$ would be equally suitable to set up the bistable mutual inhibition system.

FGF ligand-receptor dynamics have been well studied. For instance, [33, 34] contain detailed

information in tables of parameter values and detailed assumption for estimated unknown values. In [33] we find the on and off rates for FGF binding to its cell surface receptor $k_{F,\text{on}} = 0.227$ nM^{-1}min^{-1}.

To calculate Michaelis-Menten constant, K_F, for FGF binding, the phosphorylation rate of the receptor-ligand complex needs to be estimated. Using an estimate of 1-2 seconds for receptor-ligand complex phosphorylation, the Michaelis-Menten constant is

$$K_F = \frac{k_{F,\text{off}} + k_{F,2}}{k_{F,\text{on}}} = \frac{0.003 \text{ min}^{-1} + 0.02 \text{ min}^{-1}}{0.227 \text{ nM}^{-1} \text{ min}^{-1}} \approx 0.1 \text{ nM}.$$

The decay rate of FGF ligand in the PLLP is estimated using the mean half life for FGF2 in the human body, which is 7.6 hours [31]. This estimate gives

$$\delta_F = \frac{\ln 2}{456 \text{ min}} = 0.0015 \text{ min}^{-1}.$$

We can find similar information for Wnt ligand-receptor dynamics. For instance, [30] validate a mathematical model of the Wnt signalling pathway. In this paper, we find the on and off rates for Wnt ligand binding to its cell surface receptor, Frizzled, as follows: $k_{W,\text{on}} = 7.9 \times 10^4$ M^{-1} s^{-1} and $k_{W,\text{off}} = 4.7 \times 10^{-4}$ s^{-1}.

We use CXCL12a as a proxy for a the unknown Wnt ligand. In [32], the half-life of human CXCL12a is 26 minutes. This estimate gives

$$\delta_W = \frac{\ln 2}{26 \text{ min}} = 0.027 \text{ min}^{-1}.$$

The parameters F_1 and W_1 are the steady-state concentration of FGF and Wnt receptors in the absence of competition. For these parameters, we expect the number of FGF receptors to be on the order of thousands per cell. This corresponds to a concentration of approximately 1 nM, hence, we estimate $F_1 = 1$ nM. From [30], we find that there are 30 Frizzled receptors per cell and set $W_1 = 0.03$ nM. The parameters F_0 and W_0 are the concentrations at which bound FGF receptors and bound Wnt receptors reach half-max inhibition of the other type of receptor, respectively. We set $W_0 = 0.001$ nM and $F_0 = 0.1$ nM.

The Michaelis-Menten constants, K_W and K_F, can be used to scale the results from our model to compare to the equivalent biological parameters. However, finding data to estimate these parameters has proven difficult. Since these parameters are scaled out in the analysis and do not affect the results we have set them to be 1.

Estimating the diffusion rates To estimate the diffusion rates of FGF and Wnt ligand, we compare literature values and estimate the diffusion rate from molecular weight. FGF is a protein with a molecular weight of 30.7 kD. (By comparison, actin is 42 kD.) Actin diffuses at a rate of roughly 5 μm^2/s in the cytosol ([35]), and 10 times faster in water. The ratio of the diffusion coefficients would be roughly the same as the ratio of cube root of the molecular weights. Using the estimate of diffusion of actin in water as 50μm^2/s and the ratio of cube roots of the molecular weights $(42/31)^{1/3} \approx 1.1$, we find the FGF diffusion coefficient closer to $D_F \approx 55\mu\text{m}^2/\text{s} = 3300\mu\text{m}^2/\text{min}$. However, this estimate seems too fast for FGF ligand diffusion within the PLLP given the size and scale of the PLLP.

The binding to receptors and activity of FGF is known to depend on Heparan Sulfate Proteoglycans (HSPG). Not only have HSPGs been shown to regulate FGF signalling within the PLLP, but also to be an integral part of the feedback loop that organizes the PLLP. In fact, in the PLLP with mutant HSPGs, wild type signalling is disrupted [36]. Due to the binding of FGF to HSPGs, we consider an effective diffusion coefficient for FGF ligand. Let k_{on} and k_{off} be the forward and backward rates of FGF binding to HSPGs, D_H be the diffusion coefficient of FGF bound to HSPGs, and D_F the diffusion coefficient of free FGF.

$$D_{\text{effective}} = \frac{D_H k_{\text{on}} + D_F k_{\text{off}}}{k_{\text{on}} + k_{\text{off}}} = \frac{1}{\tau_H + \tau_F}(D_H \tau_H + D_F \tau_F), \tag{28}$$

where $\tau_H = \frac{1}{k_{\text{off}}}$ and $\tau_F = \frac{1}{k_{\text{on}}}$. Then the effective diffusion coefficient can be interpreted as the weighted average diffusion where the weights are the mean fractional residence times on the bound to HSPGs and freely diffusing. In this way, the diffusion coefficient of FGF within the PLLP can be regulated by binding to HSPGs. Consequently, we set $D_F = 10\mu\text{m}^2/\text{s} = 600\mu\text{m}^2/\text{min}$. Wnt ligand has a molecular weight similar to actin (41 kD), and so we again use the ratio of cube roots of the molecular weights to estimate that $D_W \approx D_F = 600\mu\text{m}^2/\text{min}$.

Supporting References

30. Kogan Y, Halevi-Tobias KE, Hochman G, Baczmanska AK, Leyns L, Agur Z. A new validated mathematical model of the Wnt signalling pathway predicts effective combinational therapy by sFRP and Dkk. Biochem J. 2012;444(1):115–125.

31. Beenken A, Mohammadi M. The FGF family: biology, pathophysiology and therapy. Nat Rev Drug Discov. 2009;8(3):235–253.

32. Kirkpatrick B, Nguyen L, Kondrikova G, Herberg S, Hill WD. Stability of human stromal-derived factor-1α (CXCL12α) after blood sampling. Ann Clin Lab Sci. 2010;40(3):257–260.

33. Nugent MA, Edelman ER. Kinetics of basic fibroblast growth factor binding to its receptor and heparan sulfate proteoglycan: a mechanism for cooperativity. Biochemistry. 1992;31(37):8876–8883.

34. Filion RJ, Popel AS. Intracoronary administration of FGF-2: a computational model of myocardial deposition and retention. American Journal of Physiology-Heart and Circulatory Physiology. 2005;288(1):H263–H279.

35. McGrath J, Tardy Y, Dewey C, Meister J, Hartwig J. Simultaneous measurements of actin filament turnover, filament fraction, and monomer diffusion in endothelial cells. Biophysical Journal. 1998;75(4):2070–2078.

36. Venero Galanternik M Marina, Kramer KL, Piotrowski T. Heparan Sulfate Proteoglycans Regulate Fgf Signaling and Cell Polarity during Collective Cell Migration. Cell Rep. 2015;10(3):414–428.
